# Bone morphogenetic protein 4 alleviates nonalcoholic steatohepatitis by inhibiting hepatic ferroptosis

**DOI:** 10.1038/s41420-022-01011-7

**Published:** 2022-04-27

**Authors:** Xingchun Wang, Bingwei Ma, Xin Wen, Hui You, Chunjun Sheng, Le Bu, Shen Qu

**Affiliations:** 1 Department of Endocrinology and Metabolism, Shanghai Tenth People’s Hospital, School of Medicine, Tongji University, Shanghai, 200072 China; 2 Thyroid Research Center of Shanghai, Shanghai, 200072 China; 3 Department of Gastrointestinal Surgery, Shanghai Tenth People’s Hospital, School of Medicine, Tongji University, Shanghai, 200072 China

**Keywords:** Metabolic disorders, Pathogenesis

## Abstract

Nonalcoholic steatohepatitis (NASH) is a state of simple steatosis that progresses to inflammation and liver injury accompanied by ferroptosis. Bone morphogenetic protein 4 (BMP4) plays an important role in adipogenesis and differentiation, as well as in hepatic steatosis and iron regulation. However, the direct impact of BMP4 on NASH remains unclear. In this study, our aim was to investigate the effect of BMP4 on NASH and its underlying mechanism. We first explored BMP4 expression in vivo in mice and patients and in vitro in HepG2 and LO2 cell lines, and then, determined whether ferroptosis occurs in NASH. Further overexpression or inhibition of BMP4 was induced to observe the effect of BMP4 on liver ferroptosis in NASH. BMP4 expression was upregulated in patients and mice with nonalcoholic fatty liver disease, and free fatty acid (FFA)-induced HepG2 and LO2 cell lines. We observed ferroptosis in high-fat diet and high-fructose diet-fed mice and FFA-induced HepG2 and LO2 cell lines. BMP4 overexpressing plasmid was constructed and the HepG2 and LO2 cells were transfected with lentivirus (oe-BMP4), or treated with exogenously added recombinant human BMP4 or BMP antagonist noggin. BMP4 suppressed the markers of hepatic steatosis, liver inflammation, and liver injury. Upregulated BMP4 expression in HepG2 and LO2 cells reduced reactive oxygen species and malondialdehyde content and relieved ferroptosis. Mechanistically, BMP4 overexpression in hepatocytes upregulated the mRNA and protein levels of glutathione peroxidase 4 (GPX4), a central regulator of ferroptosis, while exogenous inhibition of BMP4 by noggin decreased their levels. Immunoprecipitation assays demonstrated a physical interaction between BMP4 and GPX4 in HepG2 and LO2 cells, and confocal imaging confirmed colocalization of BMP4 and GPX4. Consistently, BMP4 overexpression plays an important role in NASH by increasing GPX4 expression, therefore decreasing hepatic ferroptosis. This study proposes BMP4 as a therapeutic target for preventing steatohepatitis.

## Introduction

With changes in lifestyle, the global incidence of nonalcoholic fatty liver disease (NAFLD) has been increasing annually, making it a major public health concern [1]. NAFLD is a syndrome characterized by hepatic fatty deposition, with or without inflammation, caused by factors other than excessive alcohol intake and other liver damage. NAFLD includes nonalcoholic fatty liver (NAFL), nonalcoholic steatohepatitis (NASH), liver fibrosis, and cirrhosis. NAFL is a benign disease that can be reversed through lifestyle interventions, whereas NASH may gradually progress to end-stage liver disease; 41% of patients with NASH develop liver fibrosis within 13 years, whereas 5.4% of patients with NASH develop end-stage liver disease or even hepatoma [2]. NASH has become the main cause of cryptogenic liver cancer [3]. Thus, an in-depth understanding of the molecular mechanism underlying NASH pathogenesis is of great clinical significance.

Bone morphogenetic protein 4 (BMP4) was initially confirmed to induce the formation of bone tissue and cartilage and participate in bone repair [4]. Recent studies have found that serum BMP4 levels are significantly increased in patients with obesity and metabolic syndrome (MetS) [5], but reduced after weight loss [6, 7]. BMP4 level is also associated with visceral adipose tissue [6] and plays an important role in adipogenesis [8–10] and adipocyte differentiation [11]. A recent study reported upregulated BMP4 expression in oleic acid (OA)-induced hepatic steatosis and in a mouse model of high-fat diet (HFD)-induced NAFLD, and BMP4 may alleviate hepatic steatosis [12]. However, its role in NAFLD and NASH has not been fully elucidated and the underlying mechanism remains unclear.

Ferroptosis, an iron-dependent form of nonapoptotic cell death distinct from other types of programmed cell death, was first discovered in 2012 by Stockwell et al. [13]. Iron-dependent lipid peroxidation leads to ferroptosis [14]. Ferroptosis plays a pivotal role in the pathological processes of various diseases [15–18], as well as the pathogenesis of NASH [19]. Ferroptosis is a newly discovered type of cell death characterized by the iron-dependent accumulation of reactive oxygen species (ROS) and lipid peroxidation [20, 21]. BMP4 plays a key role in iron regulation and is a powerful activator of hepcidin, which modulate iron [22, 23].

However, the role and mechanism of BMP4 in regulating ferroptosis have not yet been reported. Based on the available scientific literature, we hypothesized that BMP4 may alleviate the occurrence and development of NASH by regulating ferroptosis. We first explored BMP4 expression in patients, mice, and free fatty acid (FFA)-induced liver cells, and then, verified the role of BMP4 in NASH-related ferroptosis to further explore the mechanism of BMP4 role in NASH.

## Results

### BMP4 levels in patients with NAFLD

**Fig. 1:**
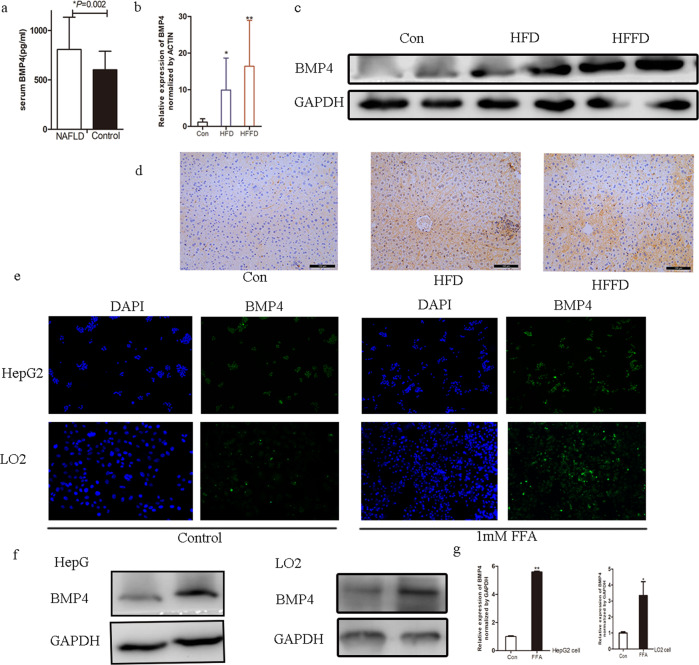
BMP4 is upregulated in human, mice with NAFLD and FFA-induced hepatocytes. **a** Serum BMP4 levels in patients with or without NAFLD, *P < 0.05. **b** Relative mRNA expression levels of BMP4 in mice with HFD, HFFD, or regular chow diet, *compared to control, *p* < 0.05, **compared to control, *p* < 0.001. **c** Western blot analysis of BMP levels in mice with HFD, HFFD, or regular chow diet. **d** Immunohistochemistry of BMP4 in mice with HFD, HFFD, or regular chow diet. **e** IF of BMP4 in FFA-induced HepG2 and LO2 cells. **f** Western blot analysis of BMP levels in FFA-induced HepG2 and LO2 cells. **g** Relative mRNA expression levels of BMP4 in FFA-induced HepG2 and LO2 cells, *compared to control, *p* < 0.05, **compared to control, *p* < 0.001.

Forty-eight patients with NAFLD (aged 46.27 ± 13.29 years) and 20 healthy age-matched individuals (42.67 ± 12.59 years) (control group) participated in this study. Serum BMP4 levels in patients with NAFLD were notably higher than those in the healthy participants (806.45 ± 327.16 vs. 601.26 ± 187.25 pg/mL, *P* = 0.002) as presented in Fig. 1a. Patients with NAFLD were divided into two groups according to the median levels of BMP4 (745.50 pg/mL). Superoxide dismutase (SOD) levels and fasting insulin (FINS) levels were significantly higher in NAFLD patients with higher BMP4 levels (*P* < 0.05). Alanine transaminase (ALT) and aspartate aminotransferase (AST) levels were slightly higher in NAFLD patients with higher BMP4 levels (*P* > 0.05). The results are presented in Table 1.

**Table 1:** Demographic and laboratory data of the patients with NAFLD.

Variable	Total (*n* = 48)	BMP < 745.50 pg/ml (*n* = 24)	BMP ≥ 745.50 pg/ml (*n* = 24)	*P* value
Age, years old	42. 67 ± 12.59	41.56 ± 11.66	43.22 ± 13.32	0.989
Gender, male/female	23/25	13/11	10/14	0.657
Weight, kg	102.77 ± 12.97	103.44 ± 12.21	102.10 ± 13.92	0.725
BMI, kg/m^2^	35.99 ± 4.27	36.33 ± 4.06	35.66 ± 4.52	0.592
TCH, mmol/l	5.36 ± 0.97	5.53 ± 0.95	5.21 ± 0.98	0.272
TG, mmol/l	1.66 (1.55,2.73)	1.66 (1.52,2.28)	1.65 (1.52,2.61)	0.523
LDL-C, mmol/l	3.23 ± 0.89	3.23 ± 0.99	3.23 ± 0.81	0.981
FFA, mmol/l	0.59 ± 0.17	0.59 ± 0.15	0.59 ± 0.19	0.994
SOD, U/ml	146.95 ± 23.00	139.32 ± 21.26	153.86 ± 22.79	0.044
ALT, U/L	38.20 (22.50,45.92)	38.60 (24.37,42.37)	34.95 (22.42,54.02)	0.930
AST, U/L	24.05 (17.30,32.65)	24.40 (17.90,30.15)	23.00 (17.10,45.40)	0.597
FBG, mmol/l	8.29 ± 3.68	7.95 ± 3.62	8.61 ± 3.78	0.560
INS, mU/L	28.10 ± 13.73	32.54 ± 13.27	23.47 ± 12.91	0.025

*NAFLD* nonalcoholic fatty liver disease, *BMI* body mass index, *TCH* total cholesterol, *TG* triglyceride, *LDL-C* low-density lipoprotein cholesterol, *FFA* free fatty acid, *SOD* superoxide dismutase, *ALT* alanine transaminase, *AST* aspartate aminotransferase, *FBG* fasting blood glucose, *FINS* fasting insulin.

### BMP4 is upregulated in FFA-induced HepG2 and LO2 cells and mouse models of fatty liver

**Fig. 2:**
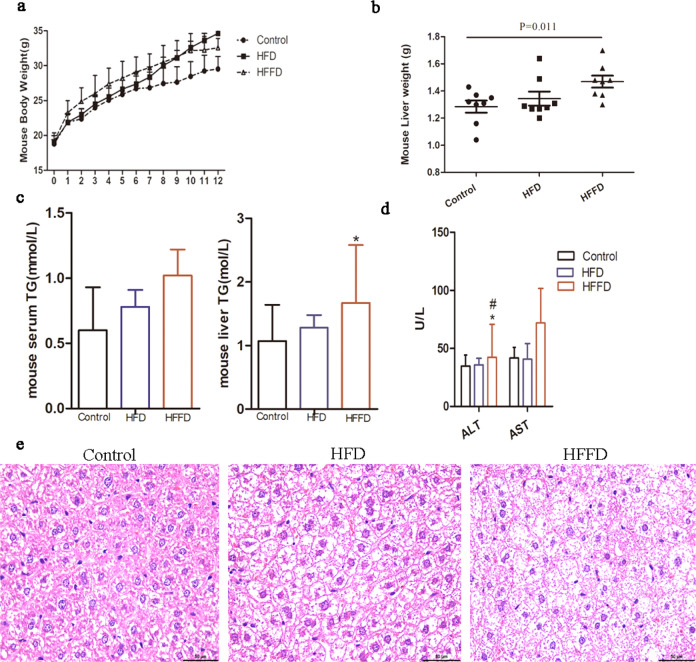
Metabolic characterization in mice. **a** Body weight in mice with HFD, HFFD, or regular chow diet. **b** Liver weight in mice with HFD, HFFD, or regular chow diet. **c** Serum and liver TG in mice with HFD, HFFD, or regular chow diet, *compared to control, *p* < 0.05. **d** Liver enzyme in mice with HFD, HFFD, or regular chow diet, *compared to control, *p* < 0.05; ^#^compared to HFD, *p* < 0.05. **e** H&E staining in mice.

Mice were fed an HFD and HFFD to establish an animal model of NAFLD to verify the expression of BMP4. Twelve weeks of HFD or HFFD led to significantly increased body weight and liver weight (Fig. 2a, b). Serum and liver triglyceride (TG) levels were significantly higher in the HFD- or HFFD-fed mice than those in the control group, as shown in Fig. 2c. The indices of liver injury, ALT, and AST were higher in the HFD- or HFFD-fed mice than those in the control group, as shown in Fig. 2d. Liver histology of H&E staining also revealed that compared to the control group, HFD- or HFFD-fed mice had a higher degree of steatosis (Fig. 2e).

It was found that the mRNA level of BMP4 was significantly increased in the liver of the animal model when compared to the control group, as shown in Fig. 1b. Additionally, western blot analysis showed that BMP4 protein was significantly upregulated in the liver of HFD- and HFFD-fed mice compared to the control mice (Fig. 1c). Immunohistochemical (IHC) results also showed that BMP4 levels were significantly upregulated in hepatic tissue of HFD- or HFFD-fed mice, as presented in Fig. 1d. In vitro, HepG2 or LO2 cells were treated with FFA, and the results of IF showed that the expression of BMP4 was remarkably increased, as shown in Fig. 1e. The mRNA and protein levels of BMP4 were also notably increased in FFA-induced HepG2 and LO2 cells, as shown in Fig. 1f, g, indicating an increase in BMP4 levels in NAFLD.

### Ferroptosis in mice with diet-induced hepatic steatosis and FFA-induced HepG2 or LO2 cells

**Fig. 3:**
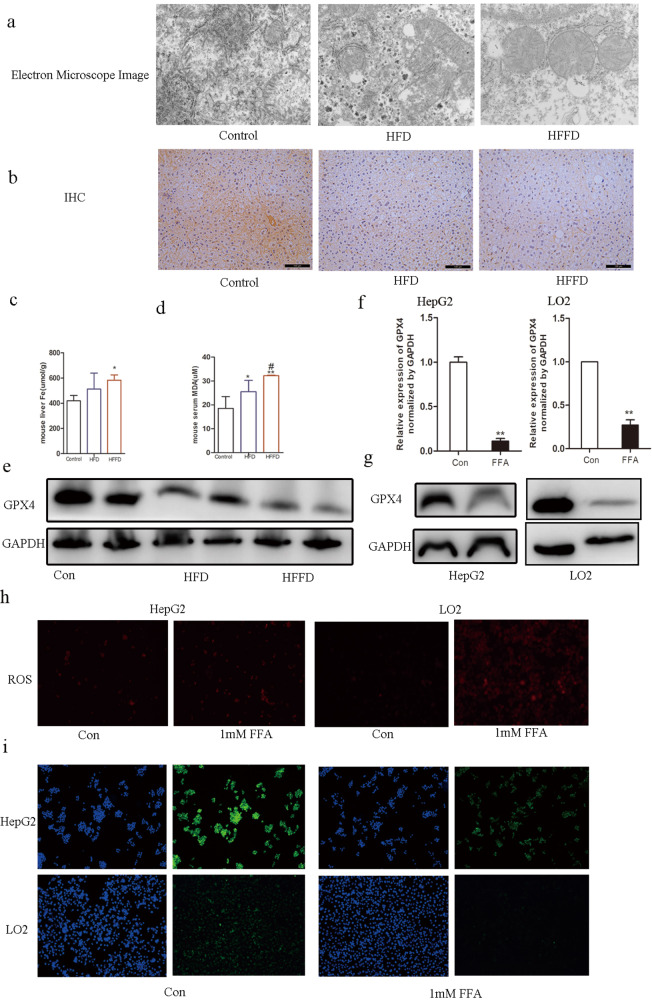
Ferroptosis in mice with NAFLD and FFA-induced hepatocytes. **a** Electron microscope image of the liver in mice with HFD, HFFD, or a regular chow diet. **b** IHC analysis of GPX4 expression of the liver in mice with HFD, HFFD, or regular chow diet. **c** Liver Fe in mice with HFD, HFFD, or regular chow diet. **d** Serum MDA in mice with HFD, HFFD, or regular chow diet. **e** Western blot analysis of GPX4 levels in mice with HFD, HFFD, or regular chow diet. **f** Relative mRNA expression levels of GPX4 in FFA-induced HepG2 and LO2 cells, **compared to control, *p* < 0.001. **g** Western blot analysis of GPX4 levels in FFA-induced HepG2 and LO2 cells. **h** ROS expression in FFA-induced HepG2 and LO2 cells. **i** IF of GPX4 expression in FFA-induced HepG2 and LO2 cells.

Glutathione peroxidase 4 (GPX4), a marker to evaluate ferroptosis, is essential for maintaining lipid homeostasis in cells, and it is negatively associated with the levels of ferroptosis [14]. Additionally, glutathione (GSH) content, lipid peroxidation, iron accumulation, and mitochondrial morphology are also critical evidence for the characterization of ferroptosis. Electron microscopy, which can observe ultrastructure, showed mitochondrial impairment in morphology, which was notably observed in HFD- or HFFD-fed mice when compared to that in the control, as shown in Fig. 3a. It was found that in IHC staining, GPX4 was notably reduced in the liver of HFD- or HFFD-fed mice when compared to the control, as shown in Fig. 3b. Fe levels were higher in HFD- or HFFD-fed mice than those in the control mice, as shown in Fig. 3c. Serum MDA levels were significantly higher in HFD- or HFFD-fed mice than those in the control mice, as shown in Fig. 3d. The protein level of GPX4 was decreased in HFD- or HFFD-fed mice when compared to that in the control, as shown in Fig. 3e. In the in vitro experiment, mRNA level of GPX4 was decreased in HepG2 or LO2 cells treated with FFA, as shown in Fig. 3f. Protein levels corresponded to the mRNA expression as GPX4 was decreased in HepG2 or LO2 cells treated with FFA, as shown in Fig. 3g. ROS level was significantly increased in HepG2 and LO2 cells treated with FFA, as shown in Fig. 3h. IF staining also showed decreased GPX4 levels in HepG2 and LO2 cells treated with FFA, as shown in Fig. 3i. These results indicate that ferroptosis is enhanced in the liver of patients with NAFLD.

### BMP4 suppresses lipid deposition, liver injury, and inflammation in FFA-induced HepG2 and LO2 cells

**Fig. 4:**
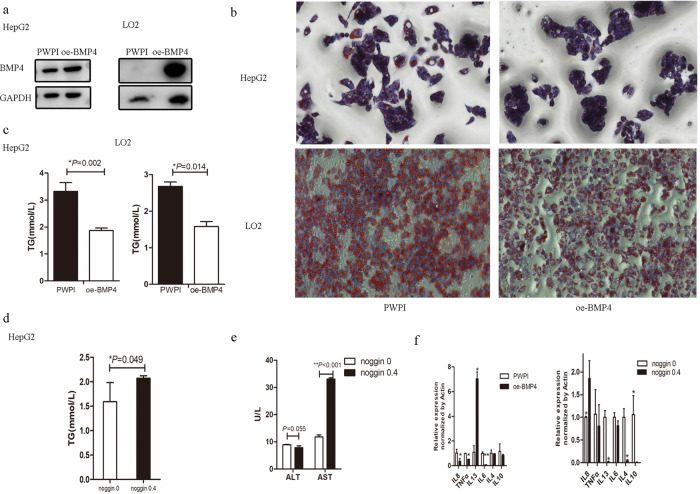
BMP4 inhibits steatohepatitis and inflammation in FFA-induced hepatocytes. **a** BMP4 overexpression vector was constructed and expressed in HepG2 and LO2 cells (oe-BMP4) after lentivirus transfection. **b** Oil red O staining after FFA intervention in oe-BMP4 and control group **c** Lipid content of TG in oe-BMP4 group and control group after FFA intervention, *p < 0.05. **d** TG content of noggin added and control group after FFA intervention, *p < 0.05. **e** Liver transaminase of noggin added and control group after FFA intervention, **p < 0.001. **f** mRNA levels of inflammatory factors in oe-BMP4 and control group. **g** mRNA levels of inflammatory factors in noggin added and control group, *p < 0.05, **p < 0.001.

To further verify the function of increased BMP4 in NAFLD, we overexpressed BMP4 (oe-BMP4) in HepG2 and LO2 cells, as shown in Fig. 4a. Endogenous overexpression of BMP4 inhibited lipid deposition in HepG2 and LO2 cells treated with FFA and tested by Oil Red O staining, as shown in Fig. 4b. Lipid deposition of TG levels was also decreased in FFA-induced HepG2 and LO2 cells with endogenous overexpression BMP4 as in Fig. 4c. However, TG levels were higher in noggin-treated HepG2 cells, as shown in Fig. 4d. Additionally, markers of liver injury and AST were significantly higher in the noggin-treated HepG2 cells, as shown in Fig. 4e. mRNA level of the pro-inflammatory cytokine, interleukin (IL)-8, IL-6, and tumor necrosis factor was significantly lower, whereas mRNA level of the anti-inflammatory cytokine, IL-13, was significantly higher in oe-BMP4 group as in Fig. 4f. mRNA level of the pro-inflammatory cytokine IL-8 was significantly higher, whereas mRNA level of the anti-inflammatory cytokine, IL-13, and IL-4, was significantly lower in the noggin group, as shown in Fig. 4f. Endogenous overexpression BMP4 inhibits lipid deposition, liver injury, and inflammation. This may affect the manifestation of NAFLD. The underlying mechanism requires further investigation.

### BMP4 suppresses ferroptosis by increasing GPX4

**Fig. 5:**
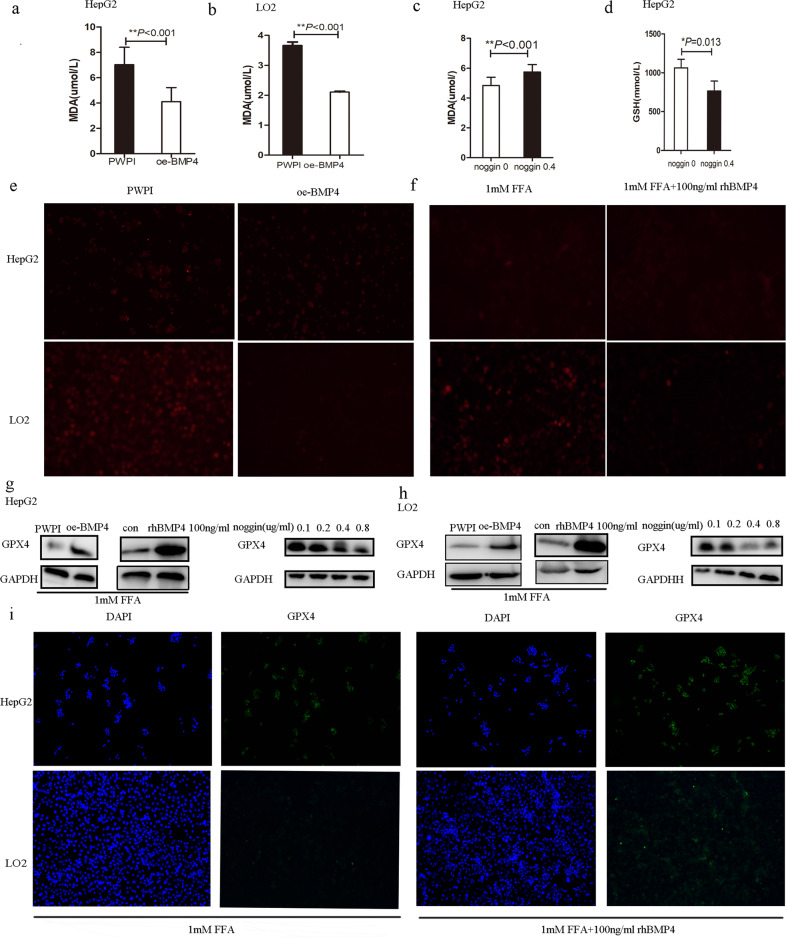
BMP4 relieves ferroptosis of NASH in FFA-induced hepatocytes. **a**, **b** MDA levels in oe-BMP4 group and control group in HepG2 and LO2 cells, **p < 0.001. **c** MDA levels in noggin added and control group in HepG2 cells, **p < 0.001. **d** GSH levels in noggin added and control group in HepG2 cells, *p < 0.05. **e** ROS expression in oe-BMP4 group and control group in HepG2 and LO2 cells. **f** ROS expression in rhBMP4 added and control group in HepG2 and LO2 cells. **g**, **h** Western blot analysis of GPX4 expression in oe-BMP4 or noggin added group and control group in HepG2 and LO2 cells. **i** IF of GPX4 expression in FFA-induced HepG2 and LO2 cells with or without rhBMP4 added.

To further explore the underlying mechanism of increased BMP4 in NAFLD, we upregulated BMP4 to examine ferroptosis in the liver. MDA levels were significantly lower in the oe-BMP4 group than in the control group of HepG2 and LO2 cells treated with FFA, as shown in Fig. 5a, b. However, MDA was significantly higher and GSH was significantly lower in HepG2 cells treated with noggin than in the control, as shown in Fig. 5c,d. ROS expression was significantly decreased in FFA-induced HepG2 and LO2 cells overexpressing BMP4 (oe-BMP4) compared to the control, as shown in Fig. 5e. Additionally, FFA-induced HepG2 and LO2 cells with recombinant human BMP4 (rhBMP4) also showed decreased ROS levels when compared to the control, as shown in Fig. 5f. Further exploration of the effect of BMP4 on GPX4, a core marker of ferroptosis, showed that FFA-induced HepG2 cells had higher expression of GPX4 protein in the oe-BMP4 group than those in the control, while FFA-induced HepG2 cells had lower expression of GPX4 protein in the noggin group than those in the control, as shown in Fig. 5g. The effect of BMP4 on GPX4 protein was also observed in LO2 cells, as shown in Fig. 5h. IF measurement also verified the results as an expression of GPX4 was increased in FFA-induced HepG2 and LO2 cells overexpressing BMP4 (oe-BMP4 or rhBMP4), as shown in Fig. 5i. BMP4 plays a role in ferroptosis, partly by regulating the expression of GPX4.

### BMP4 interacted with GPX4

**Fig. 6:**
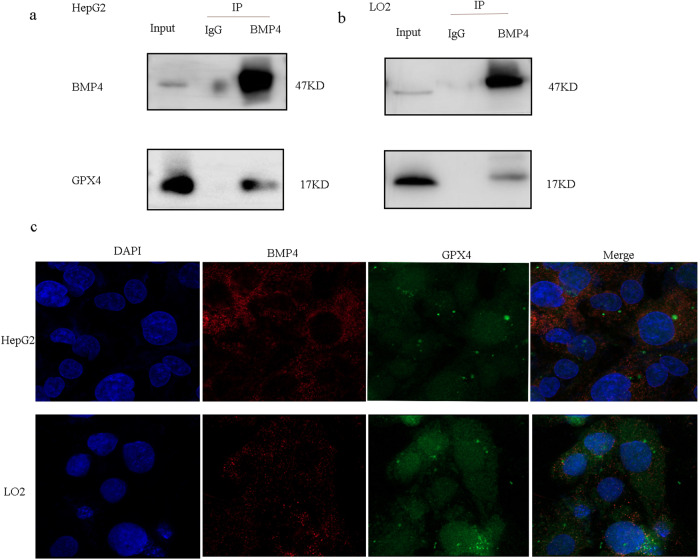
BMP4 interacted with GPX4. **a** IP analysis demonstrated that BMP4 protein in HepG2 and LO2 cells interacted with GPX4. **b** Immunofluorescence analysis suggested that GPX4 and BMP4 were co-located.

Immunoprecipitation (IP) experiments demonstrated a physical interaction between BMP4 and GPX4 using IP assays with BMP4 antibodies in HepG2 and LO2 cells (Fig. 6a, b). These data suggest that BMP4 may activate GPX4 through a non-transcriptional mechanism. Additionally, representative confocal imaging of HepG2 cells and LO2 cells showed the colocalization of BMP4 and GPX4 in HepG2 and LO2 cells, as shown in Fig. 6c.

## Conclusion

BMP4 levels are increased in NAFLD and negatively correlated with lipid deposition, liver injury, and inflammation in NASH; upregulated BMP4 expression may suppress NASH-related ferroptosis. Mechanistically, the effect of BMP4 on ferroptosis may be mediated by modulating GPX4 expression. Thus, BMP4 may be exploited as a new therapeutic target for patients with NASH.

## Materials and methods

### Participants

Participants were recruited from the Endocrine and Metabolism Department of the Shanghai Tenth People’s Hospital. A total of 48 patients with NAFLD were included, and 20 age-matched individuals were used as controls. Hepatic steatosis was assessed using FibroScan, which is a non-invasive method for evaluating hepatic steatosis [24]. We used 288 db/m as the threshold for diagnosing NAFLD [25]. The participants included in this study had no history of alcohol consumption or their alcohol intake was less than 70 g/week in males and less than 140 g/week in females. In addition, those with viral hepatitis and autoimmune liver disease were excluded. All participants provided signed informed consent. This study was approved by the Ethical Committee of the Shanghai Tenth People’s Hospital. Anthropometric measurements (height and body weight) and laboratory measurements (total cholesterol, TG, low-density lipoprotein cholesterol, FFA, SOD, ALT, AST, fasting blood glucose, FINS) were obtained. Additionally, serum BMP4 levels were measured using human enzyme-linked immunosorbent assay kits (ab99982, Abcam). Every serum was replicated twice and the average was adopted for analysis.

### Mice models of steatohepatitis

Male C57BL/6 J mice (6–8 weeks old) were purchased from the Shanghai Laboratory Animal Center (Shanghai, China). All mice used in our study were housed in standard cages and maintained in a controlled room with 12:12 h light–dark at 22 °C (Animal House, Shanghai Tenth People’s Hospital of Tongji University). They were provided with water ad libitum and 40% ± 5% relative humidity. The study protocol was approved by the Animal Care and Use Committee of Tongji University. In all, 30 male mice were blindly randomly divided into three groups. Chow-fed animals (*n* = 10) were provided a regular chow diet, HFD cohort (*n* = 10) was fed a diet with 60% kcal from fat (Medicience Ltd., Jiangsu, China), and the high-fructose diet (HFFD)(*n* = 10) cohort was given a diet with 60% high-fat and 10% fructose (Medicience Ltd., Jiangsu, China) for 12 weeks.

### Quantitative real-time reverse-transcriptase polymerase chain reaction (qRT-PCR) analysis

Total mRNA was isolated from the mouse liver using TRIzol reagent (Invitrogen/Ambion 15596-026). CDNA preparation and qRT-PCR analyses were carried out according to protocols provided by the manufacturers of the kits. PrimeScript RT Reagent Kit (TaKaRa, Japan) was used for RT-PCR. qRT-PCR was performed using a 7900H T real-time PCR system (ABI, CA, USA) to determine the expression of target genes according to the instructions of SYBR Green Master Mix (KAPA Japan) for quantitative PCR. Every sample was replicated three times. Glyceraldehyde 3-phosphate dehydrogenase (GAPDH) or ACTIN was used as an endogenous control for gene expression analysis using the ΔΔCt method. Data are presented as fold change over control, and the ratio of the mRNA expression level of the target gene to GAPDH or ACTIN was 2−△C (T), where △C (T) = C (T) target gene–C (T) GAPDH or ACTIN. Primer sequences are available on request (Table 2).

**Table 2:** Primer sequences for qRT-PCR.

Genes	Forward primer sequence	Reverse primer sequence
homo-BMP4	AGCTTCCACCACGAAGAACAT	AAGCCCCTTTCCCAATCAGG
homo-GPX4	TTCCCGTGTAACCAGTTCGG	GCCCTTGGGTTGGATCTTCA
homo-GAPDH	AGGTCGGTGTGAACGGATTTG	TGTAGACCATGTAGTTGAGGTCA
homo-IL-8	TTTTGCCAAGGAGTGCTAAAGA	AACCCTCTGCACCCAGTTTTC
homo-TNFα	ACATACTGACCCACGGCTC	CCTGCCACGATCAGGAAGG
homo-IL-13	CCTCATGGCGCTTTTGTTGAC	TCTGGTTCTGGGTGATGTTGA
homo-IL-6	ACTCACCTCTTCAGAACGAATTG	CCATCTTTGGAAGGTTCAGGTTG
homo-IL-4	CCAACTGCTTCCCCCTCTG	TCTGTTACGGTCAACTCGGTG
homo-IL10	CTCAGCACTGCTCTGTTGC	ACAAGTTGTCCAGCTGATCC
mus-ACTIN	GGCACCACACCTTCTACAATGA	ACGCTCGGTCAGGATCTTCA
mus-BMP4	AGCCAACACTGTGAGGAGTT	ATACGGTGGAAGCCCTGTTC
mus-GPX4	ACGAATTCTCAGCCAAGGACA	ACGAATTCTCAGCCAAGGACA

*qRT-PCR* real-time reverse-transcriptase polymerase chain reaction, *BMP4* bone morphogenetic protein 4, *GPX4* glutathione peroxidase 4; *IL* interleukin; *TNF* tumor necrosis factor.

### Western blotting

Western blotting was performed according to a previously described procedure [26]. The following antibodies were used to test the expression of protein: anti-BMP4 (EPR6211) (ab124714, Abcam), anti-BMP4 (ab39973, Abcam), and anti-glutathione peroxidase 4 (anti-GPX4) (ab125066, Abcam). Anti-GAPDH (#5174, Cell Signaling Technology) was used as an internal loading control. Experiments were repeated more than three times, and representative images are presented in the article at the end.

### Immunofluorescence (IF) and confocal fluorescence microscopy

HepG2 and LO2 cells were washed with phosphate-buffered saline (PBS) twice and fixed with 4% paraformaldehyde (PFA) for 18 min. The cells were washed with PBS twice and blocked for 60 min with 3% bovine serum albumin, and permeabilized with 0.2% Triton-100X before incubation with the primary antibody. The primary antibody against BMP4 and GPX4 was diluted 500-fold and incubated at 4 °C overnight. Cells were washed with PBS three times and incubated with goat anti-rabbit IgG H&L secondary antibody (ab150077, Abcam) at 37 °C for 2 h. After washing with PBS three times, the nuclei were stained with 49, 6-diamidino-2-pheny-lindole (C1002, Beyotime Biotechnology) for 20 min. Fluorescence analysis was performed using a laser scanning microscope (Leica). Additionally, HepG2 and LO2 cells were stained with both BMP4 and GPX4 antibodies. Then, images were obtained with a confocal fluorescence microscope (Leica TSC-SP2 HCX PL APO, ×63/1.32–0.60 oil objective) and acquired using the Leica Confocal Software W (Leica Microsystem, Wetzlar, Germany).

### Histology and IHC staining

The retrieved mouse liver samples were fixed with 4% PFA. They were then embedded in paraffin and sectioned. The sections were deparaffinized, rehydrated, and subjected to hematoxylin and eosin (H&E) staining and IHC staining, as described. The sections were deparaffinized for H&E and IHC staining. IHC staining was followed by antigen retrieval and immunostaining with anti-BMP4 and GPX4 antibodies. HepG2 and LO2 cells were stained with Oil Red O to quantify the lesion sizes. For immunostaining, HepG2 and LO2 cells were incubated first with cleaved BMP4 and GPX4 antibodies and subsequently, with secondary antibodies.

### Measurement of hepatic injury, TG, and ferroptosis

The serum and hepatic tissue injury markers, ALT and AST, were measured according to the manufacturer’s instructions (C009-2-1 and C010-2-1, Nanjing Jiancheng). TG was measured using the GPO-PAP method (A110-1-1, Nanjing Jiancheng). The tissue of Fe in mice was measured by a Fe kit using a colorimetric method (A039-2-1, Nanjing Jiancheng). Markers of ferroptosis were tested as follows: malondialdehyde (MDA) was tested using an MDA Assay Kit (S0131S, Beyotime Biotechnology), and ROS was tested using the ROS Assay Kit (E004-1-1, Nanjing Jiancheng).

### Electron microscopy

Liver samples were fixed in 2.5% glutaraldehyde (pH 7.4) for 2 h. They were washed three times with 0.1 M phosphate buffer (pH 7.2) and fixed in 1% osmic acid at 4 °C for 2 h. The samples were then dehydrated in a graded series of ethanol after three washes with ddH_2_O. Subsequently, the samples were embedded in Epon-Araldite resin for penetration and placed in a model for polymerization at a temperature of 60 °C. A semi-thin section was used for positioning, and an ultrathin section was prepared and collected for microstructure analysis using Leica UC7. Counterstaining was performed using 3% uranyl acetate and 2.7% lead citrate. The sections were observed using an HT7800 transmission electron microscope.

### Immunoprecipitation

For IP experiments, HepG2 and LO2 cells were lysed using radioimmunoprecipitation assay (RIPA) buffer containing protease inhibitors, cleared by centrifugation, and the protein concentration was estimated. Lysates (500 μg) were precleared with Protein A/G beads. Pre-adsorption for 30 min, followed by centrifugation for 15 min was performed. The supernatant was collected, and BMP4 (Abcam, ab124715) antibody was added to the supernatant, and then immunoprecipitated overnight at 4 °C. The antigen and antibody mixture was incubated in Protein A/G for 4 h. After centrifugation and discarding the supernatant, 1 mL RIPA lysis buffer was added, followed by centrifugation (repeat 3–5 times), the supernatant was discarded, and a loading buffer was added. Western blotting was performed as described previously.

### Cell cultures

HepG2 and LO2 cells (obtained from Shanghai Tenth People’s Hospital of Tongji University) were maintained in Dulbecco’s Modified Eagle Medium (DMEM) (Gibco) containing 10% fetal bovine serum (Gibco) and 1% penicillin-streptomycin (Gibco). The cells were incubated in a humidified atmosphere of 5% CO_2_ at 37 °C. Cell lines were tested and found to be free of mycoplasma. OA and palmitic acid (PA) can induce lipid accumulation [27]. Three hundred microliters of OA [40 mM]) and PA (20 mM) (2:1 concentration configuration) were added to 8400 µL DMEM to get 1 mM FFA. Active rhBMP4 (ab238298, Abcam) and BMP antagonist noggin (ab73756, Abcam) were added to cells to observe the effect of BMP4 on hepatic cells.

### In vitro overexpression of BMP4

The lentivirus vector control was constructed as the control sequence and it was delivered into cells under the same conditions, whereas the lentivirus vector BMP was the interference sequence (Genomeditech Biotechnology Co. Ltd, Shanghai, China). Western blotting was used to validate the efficiency of BMP4 overexpression.

### Statistical analysis

Data were analyzed using SPSS 20.0 statistical software. Normally distributed continuous data are presented as the mean ± standard deviation (X ± SD). Categorical variables are presented as numbers. Two-tailed Student’s *t* test was used to compare normally distributed data. Non-normally distributed continuous data were compared using the Mann–Whitney *U* test. Statistical significance was set at *P* < 0.05. Additionally, according to the mean value and standard deviation of NAFLD and Non-NAFLD group, the test power can reach 0.94 through Power Analysis and Sample Size software (version 11) to assess the test power of the current sample size.

## Discussion

BMP4 plays an important role in the development of metabolic diseases. A previous study showed that serum BMP4 levels are significantly increased in individuals with obesity or MetS and positively correlated with body mass index, waist circumference, and waist-to-hip ratio. Increased BMP4 expression regulates both white and beige adipogenic commitment and differentiation. The liver, the main site of ectopic fat deposition was also affected by BMP4. Previous studies have found that BMP4 alleviates hepatic steatosis, promotes hepatic glycogen accumulation, and reduces glucose levels [12, 28]. It may be a new therapeutic target for the targeted therapy of NAFLD, even in the progression of NAFLD, including NASH. However, no study has focused on the study of BMP4 on NASH, which is the development of hepatic steatosis.

To verify the effect of BMP4 on NAFLD, we first measured the levels of serum BMP4 in patients with NAFLD. The results indicated that serum BMP4 levels in patients with NAFLD were notably higher than those in healthy participants. Additionally, the preliminary results indicated that BMP4 expression was upregulated in the livers of HFD- or HFFD-induced mice and FFA-treated hepatocytes, in accordance with the results observed in humans. To further verify the effects of increased BMP4 levels on NASH progression, the NASH phenotype was tested by overexpressing BMP4 or adding exogenous rhBMP4 to FFA-treated hepatocytes. The results showed that BMP4 relieved hepatic lipid deposition, which was consistent with the findings of a previous study [12]. We also observed BMP4 role in relieving liver injury and inflammation. These results suggested that BMP4 may be involved in relieving NAFLD as well as mitigating NASH progression.

Ferroptosis, a type of regulated cell death, is manifested by elevated iron levels and excessive accumulation of lipid peroxidation-induced ROS. GPX4 is a key upstream regulator of ferroptosis [29]. It is a repair enzyme that protects the cell membrane against the peroxidation of polyunsaturated fatty acids [14, 30]. It can eliminate lipid peroxides by catalyzing GSH to oxidize GSH disulfide. Therefore, decreased GPX4 activity can lead to ferroptosis. In our study, we observed reduced GPX4 levels and increased Fe content in HFD- or HFFD-fed mice. Furthermore, smaller mitochondria, higher membrane density, and reduced mitochondrial cristae were observed in the livers of HFD- or HFFD-fed mice. In addition, levels of ROS and MDA, the markers of ferroptosis, were increased and the expression of GPX4 was notably reduced in FFA-treated hepatocytes, which was in accordance with the results in mice. Collectively, the results of in vivo and in vitro experiments suggested that ferroptosis is activated in NASH.

BMP4 belongs to the BMP family of proteins involved in iron homeostasis [22, 23]. BMP4 is a potent activator of hepcidin, which regulates iron content. However, to the best of our knowledge, none of the studies have proved the effect of BMP4 on ferroptosis. Here, we focused on whether BMP4 overexpression plays a role in liver ferroptosis. We observed that BMP4 may regulate NASH pathogenesis, and further investigated whether BMP4 played a role in ferroptosis to regulate NASH. BMP4 overexpression in FFA-induced hepatocytes upregulated GPX4 expression, the regulatory factor of ferroptosis, at both the mRNA and protein levels. Additionally, the molecular markers of ferroptosis, ROS, and MDA contents, were downregulated with BMP4 overexpression. Taken together, these results provided evidence that BMP4 overexpression in NASH may be a protective factor against ferroptosis.

Furthermore, a physical interaction between BMP4 and GPX4 was observed in HepG2 and LO2 cells, and confocal imaging confirmed the colocalization of BMP4 and GPX4 in HepG2 and LO2 cells. Therefore, BMP4 may interact with GPX4 through nonclassical pathways, thereby regulating ferroptosis, both directly as well as indirectly.

## Supplementary information

western blot

western blot

western blot

western blot

western blot

western blot

western blot

western blot

western blot

western blot

western blot

western blot

western blot

western blot

western blot

western blot

western blot

western blot

western blot

western blot

western blot

western blot

western blot

western blot

western blot

western blot

western blot

western blot

western blot

western blot

western blot

confocal

confocal

confocal

confocal

confocal

confocal

confocal

confocal

western blot

## Data Availability

The data of this study are available from the corresponding author upon reasonable request.
