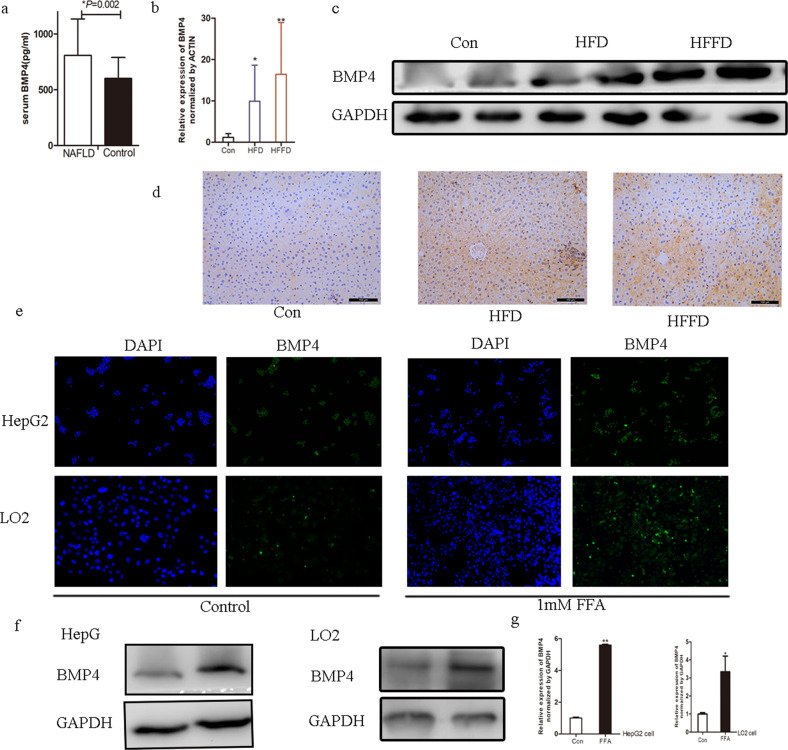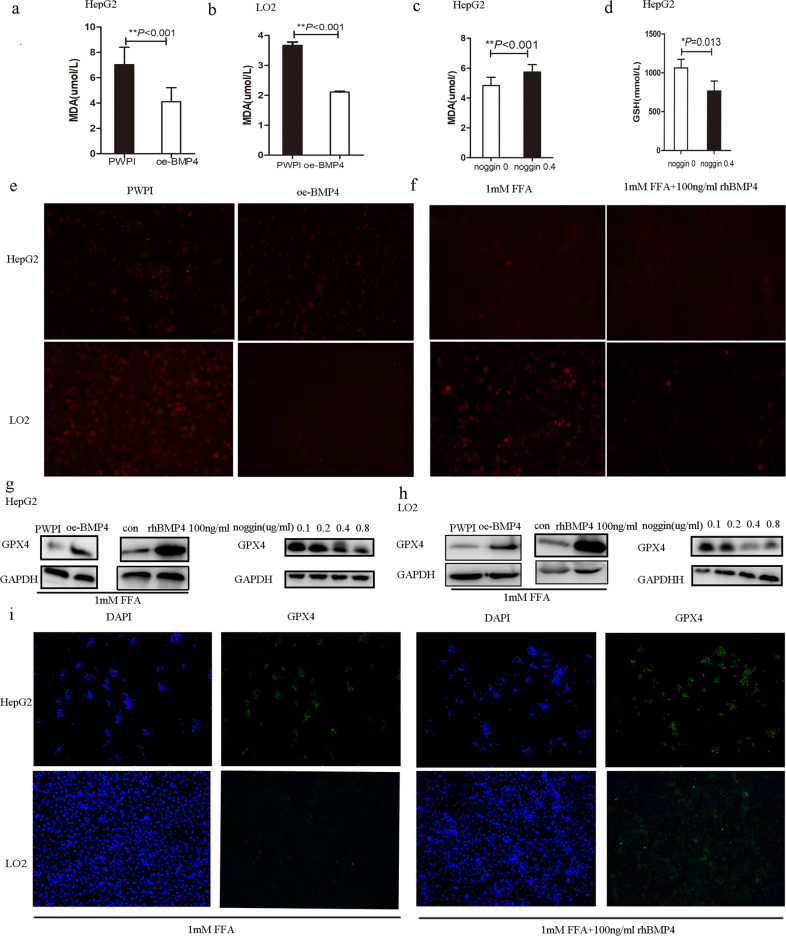# Correction: Bone morphogenetic protein 4 alleviates nonalcoholic steatohepatitis by inhibiting hepatic ferroptosis

**DOI:** 10.1038/s41420-022-01246-4

**Published:** 2022-11-24

**Authors:** Xingchun Wang, Bingwei Ma, Xin Wen, Hui You, Chunjun Sheng, Le Bu, Shen Qu

**Affiliations:** 1 Department of Endocrinology and Metabolism, Shanghai Tenth People’s Hospital, School of Medicine, Tongji University, Shanghai, 200072 China; 2 Thyroid Research Center of Shanghai, Shanghai, 200072 China; 3 Department of Gastrointestinal Surgery, Shanghai Tenth People’s Hospital, School of Medicine, Tongji University, Shanghai, 200072 China

**Keywords:** Metabolic disorders, Pathogenesis

Correction to: *Cell Death Discovery* 10.1038/s41420-022-01011-7, published online 27 April 2022

The original version of this article unfortunately contained mistakes in figures 1 and 5. The authors apologize for the errors. The correct figures can be found below. The original article has been corrected.