# Supplementary figures and images for: Bone morphogenetic protein 4 alleviates nonalcoholic steatohepatitis by inhibiting hepatic ferroptosis
Source: Cell Death Discov. 2022 Apr 27;8:234. doi: 10.1038/s41420-022-01011-7 (PMC9046379; doi:10.1038/s41420-022-01011-7)

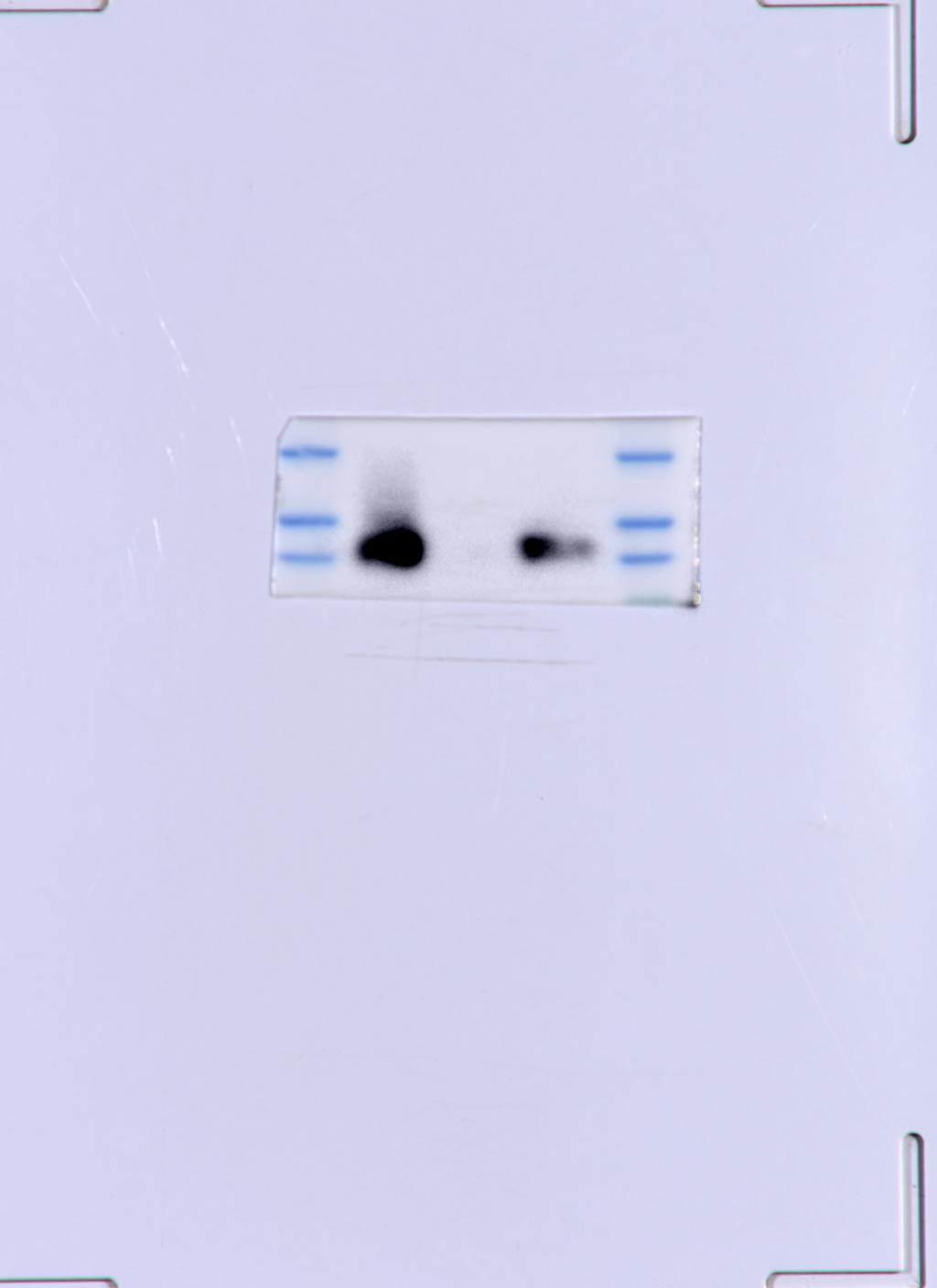

Supplement: Supplementary file 1 — western blot [file 41420_2022_1011_MOESM1_ESM.jpg]

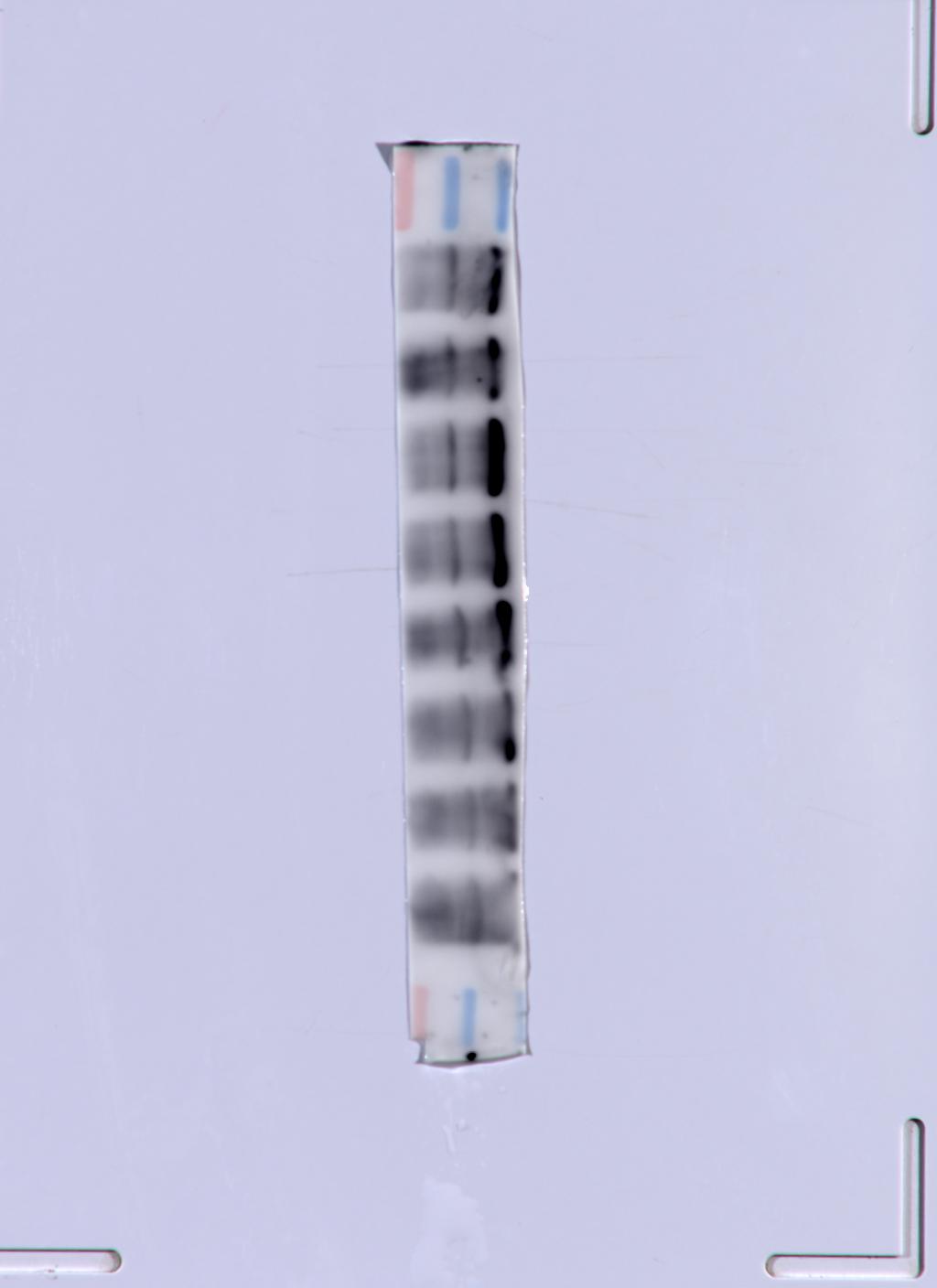

Supplement: Supplementary file 2 — western blot [file 41420_2022_1011_MOESM2_ESM.jpg]

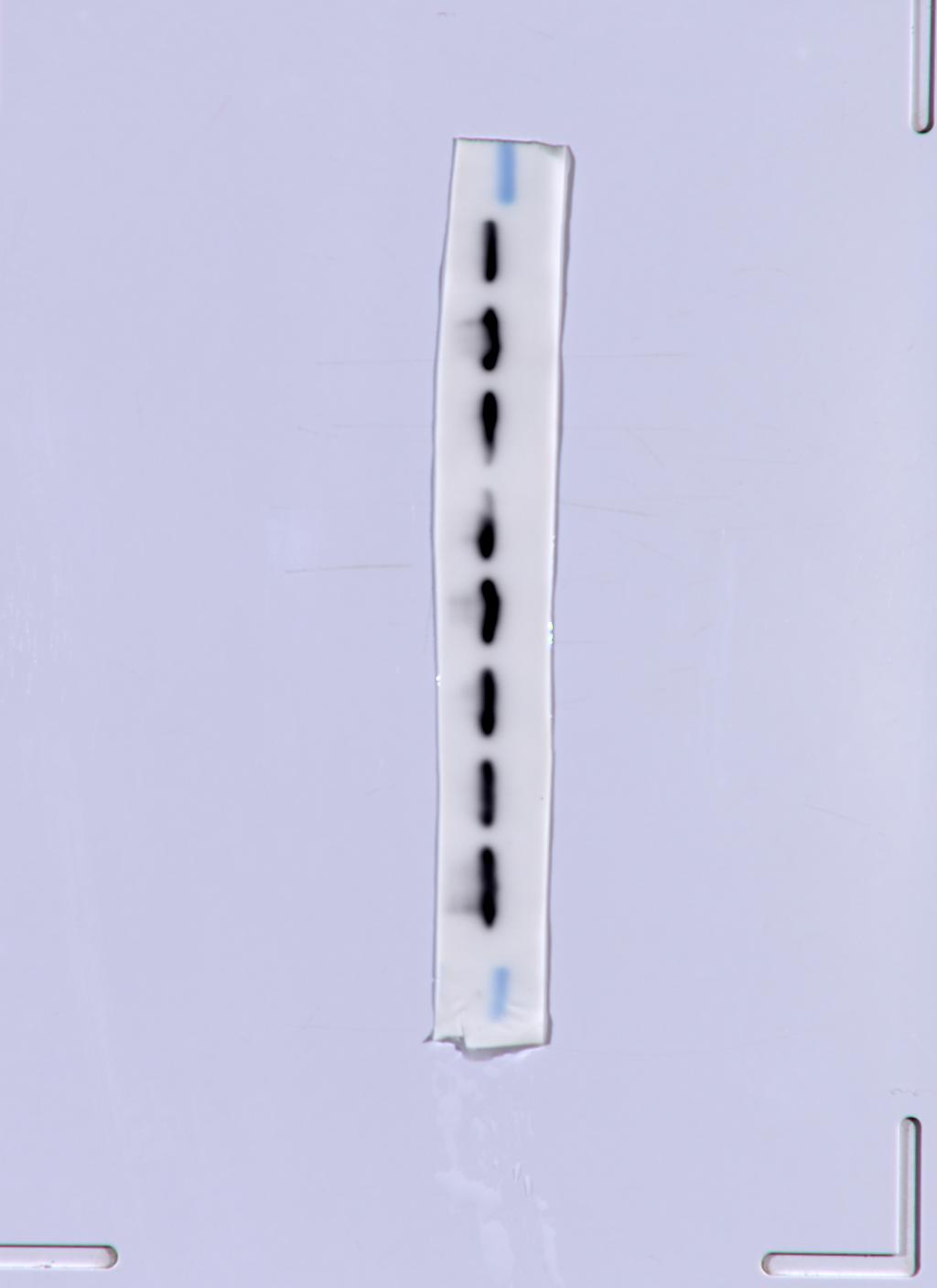

Supplement: Supplementary file 3 — western blot [file 41420_2022_1011_MOESM3_ESM.jpg]

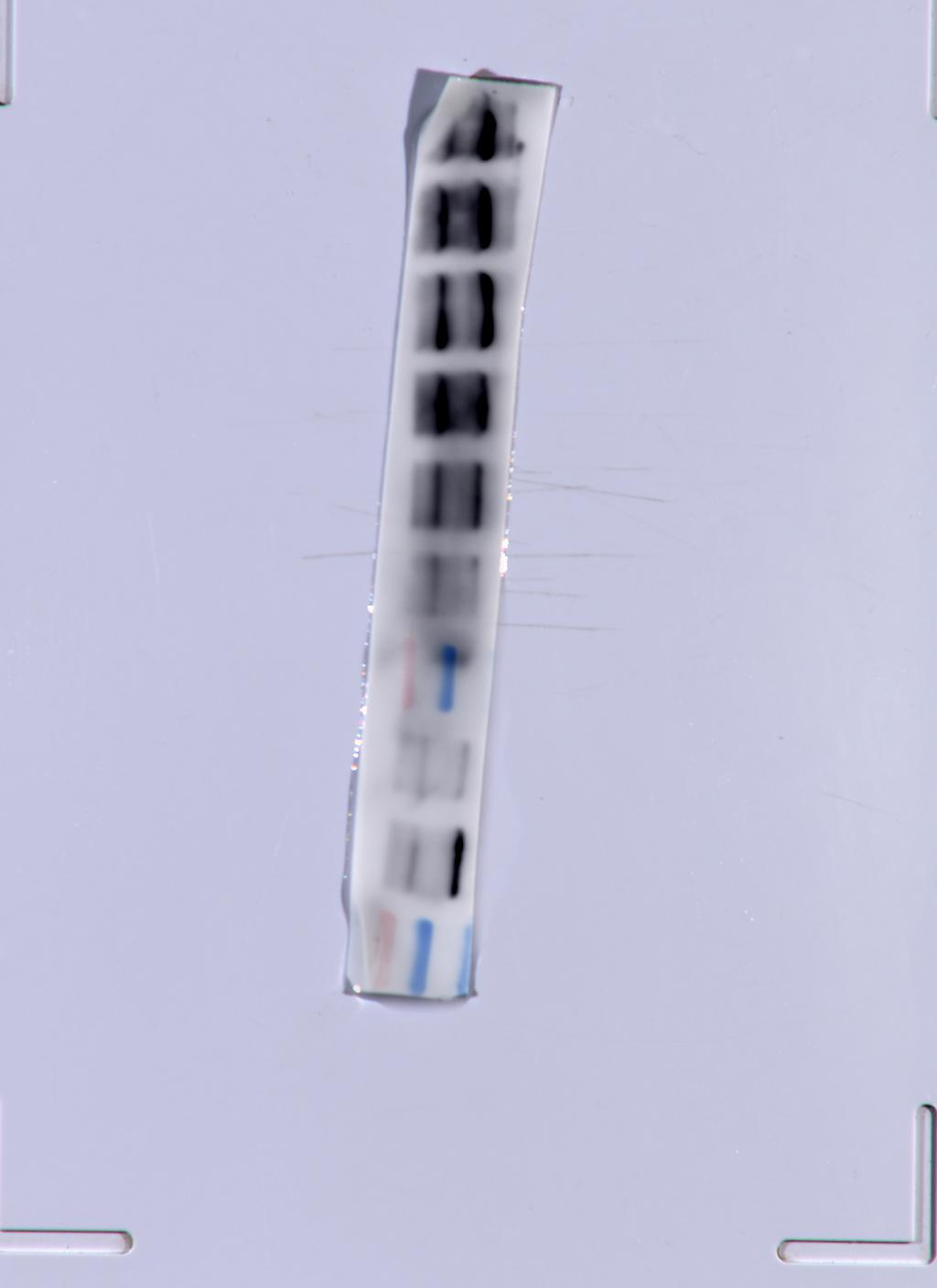

Supplement: Supplementary file 4 — western blot [file 41420_2022_1011_MOESM4_ESM.jpg]

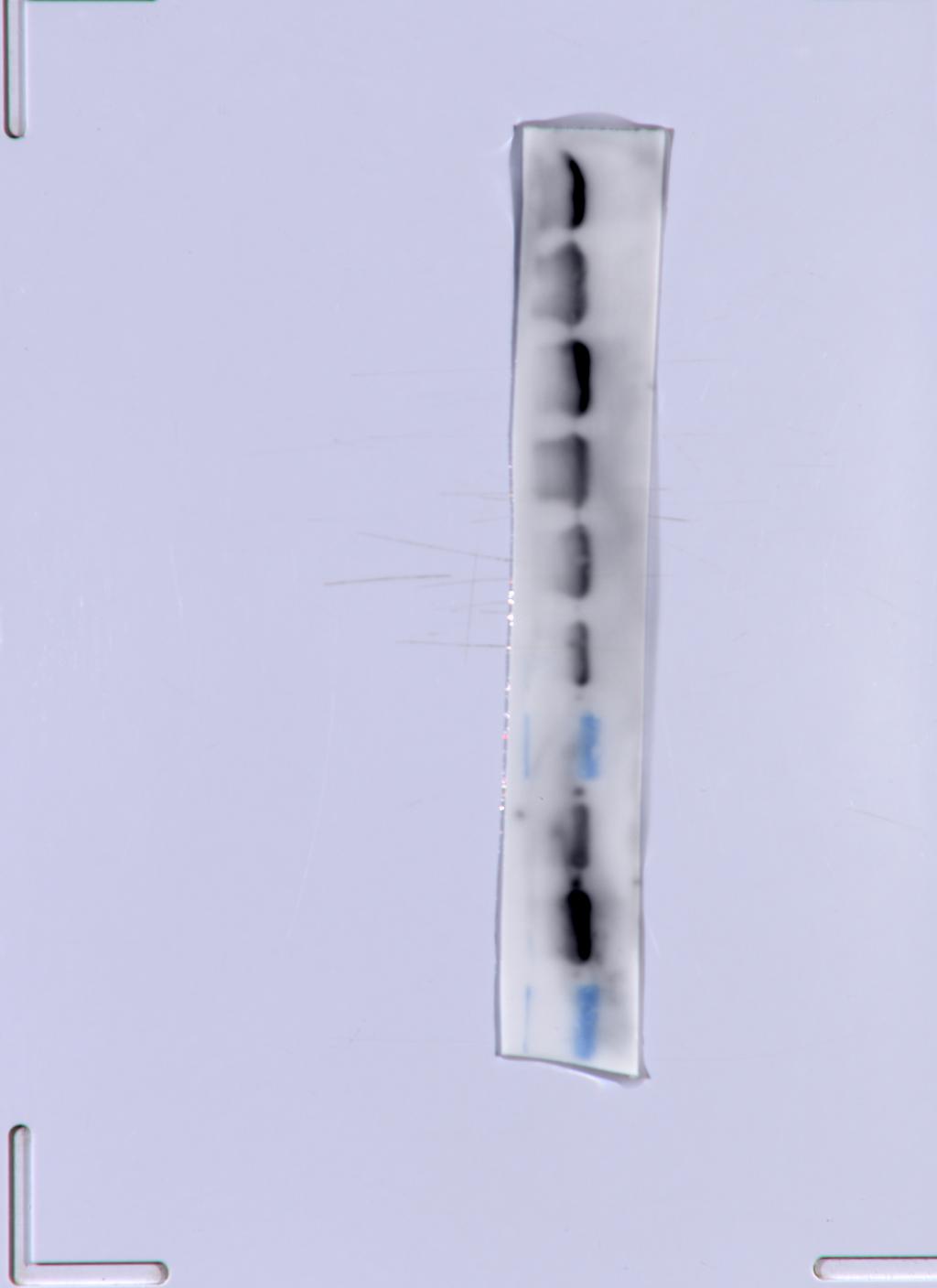

Supplement: Supplementary file 5 — western blot [file 41420_2022_1011_MOESM5_ESM.jpg]

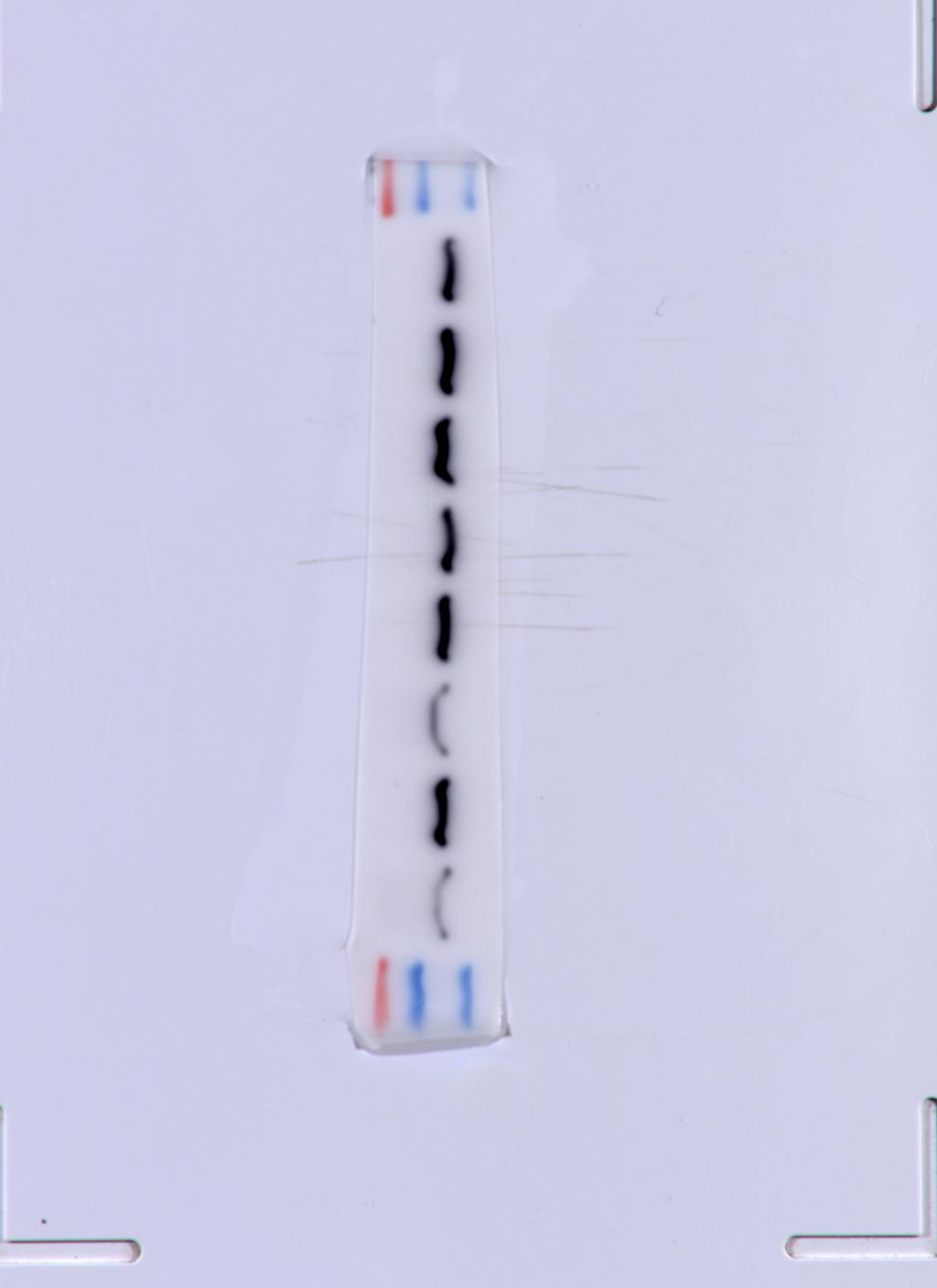

Supplement: Supplementary file 6 — western blot [file 41420_2022_1011_MOESM6_ESM.jpg]

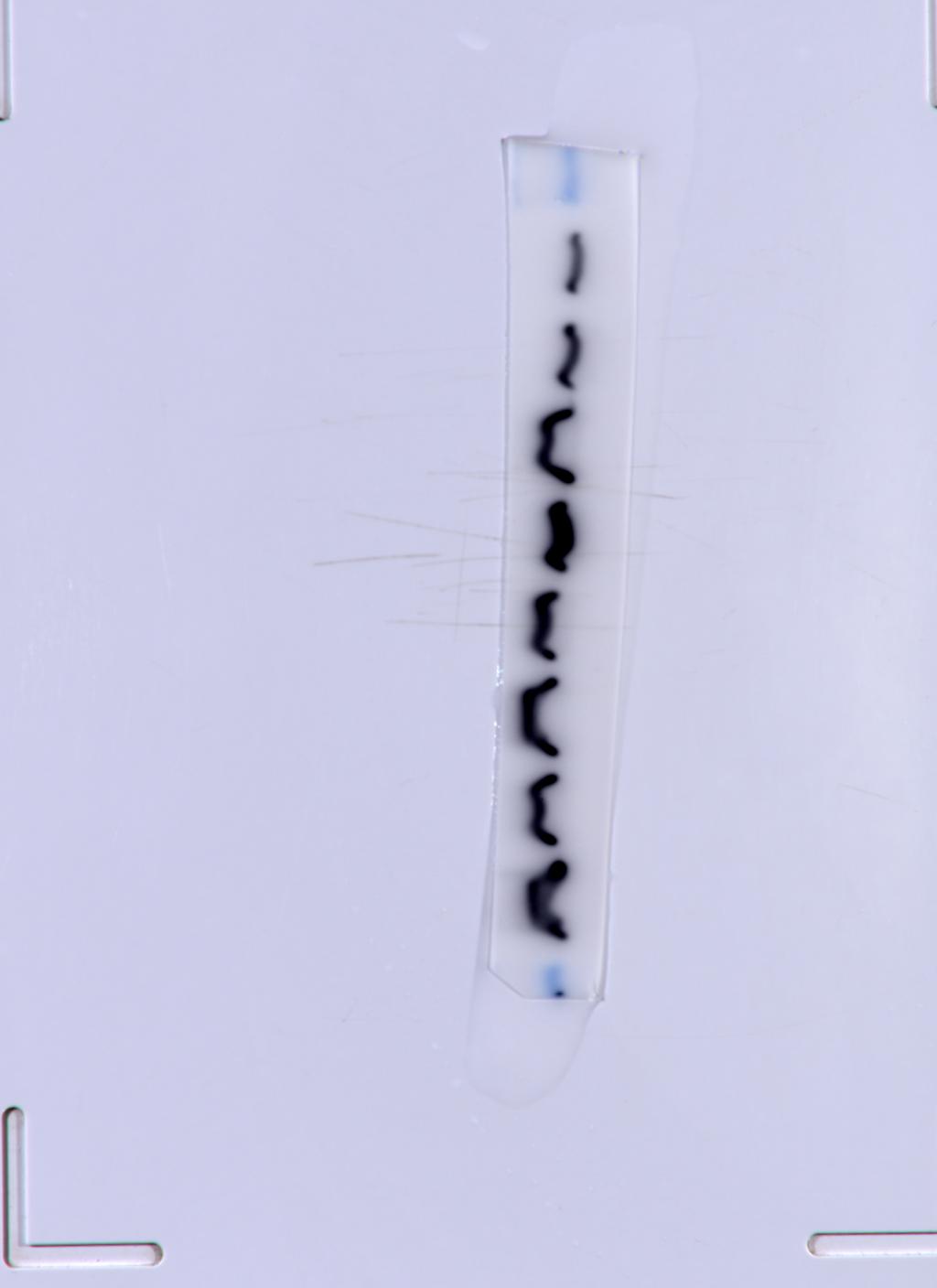

Supplement: Supplementary file 7 — western blot [file 41420_2022_1011_MOESM7_ESM.jpg]

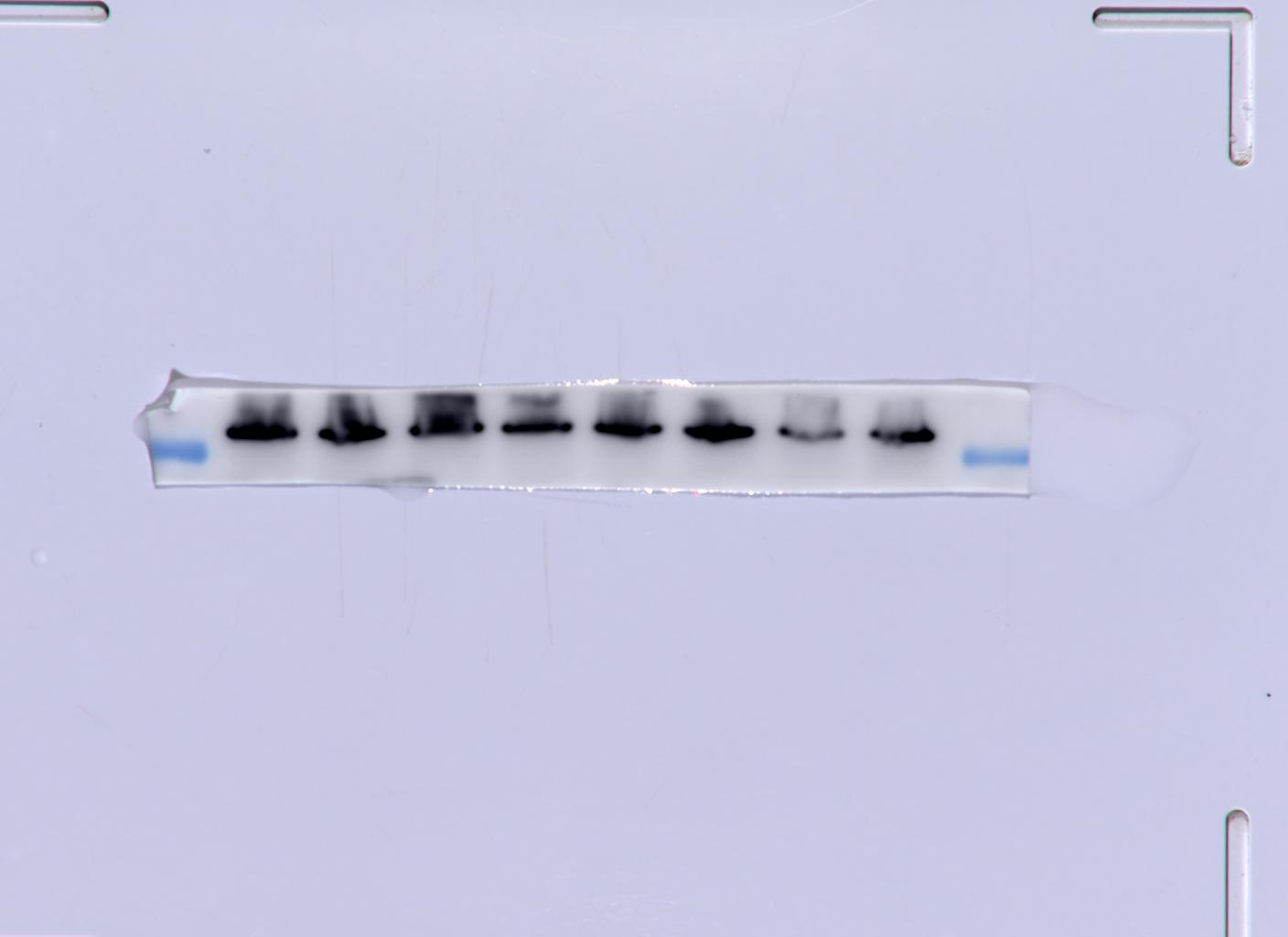

Supplement: Supplementary file 8 — western blot [file 41420_2022_1011_MOESM8_ESM.jpg]

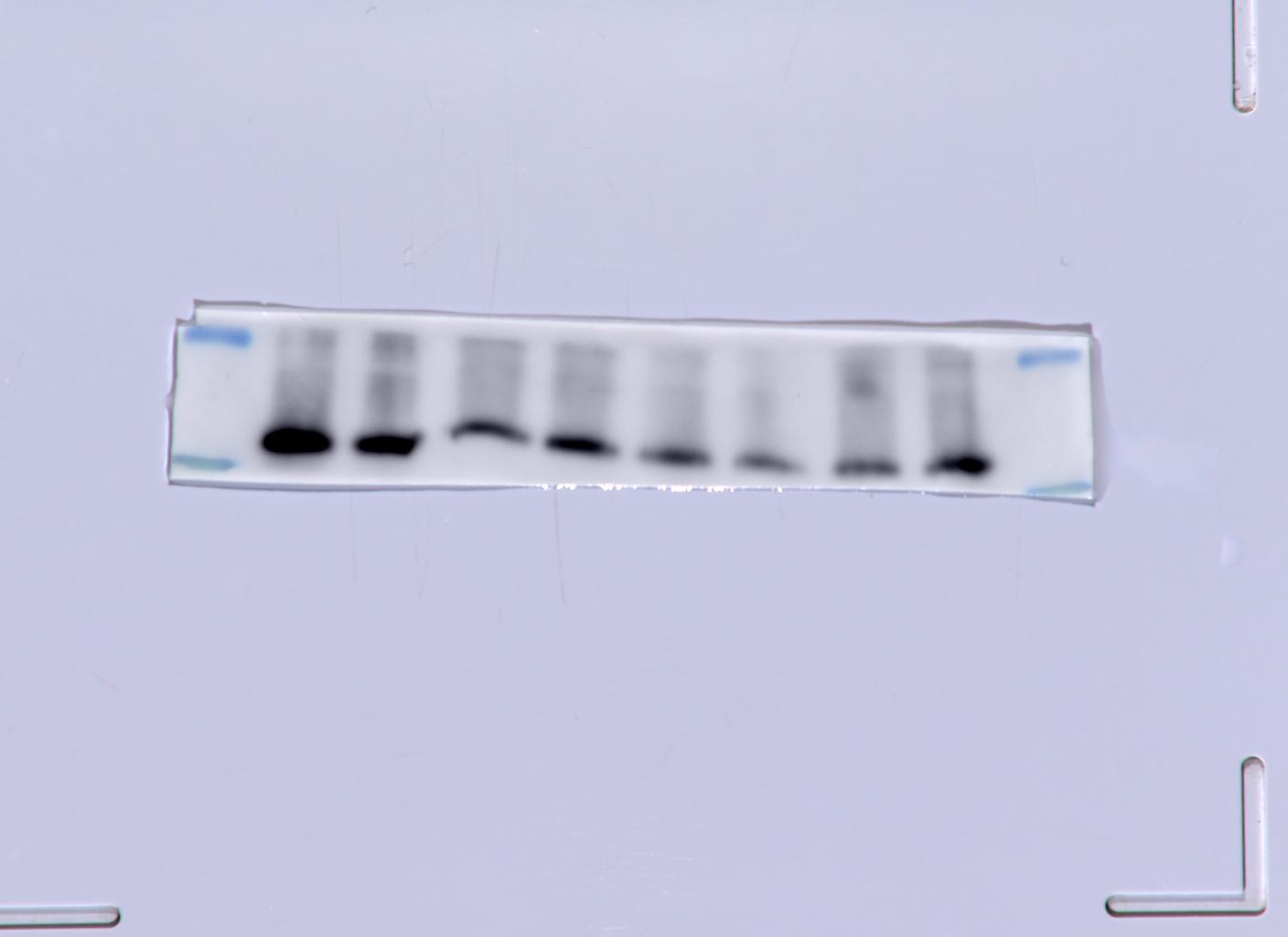

Supplement: Supplementary file 9 — western blot [file 41420_2022_1011_MOESM9_ESM.jpg]

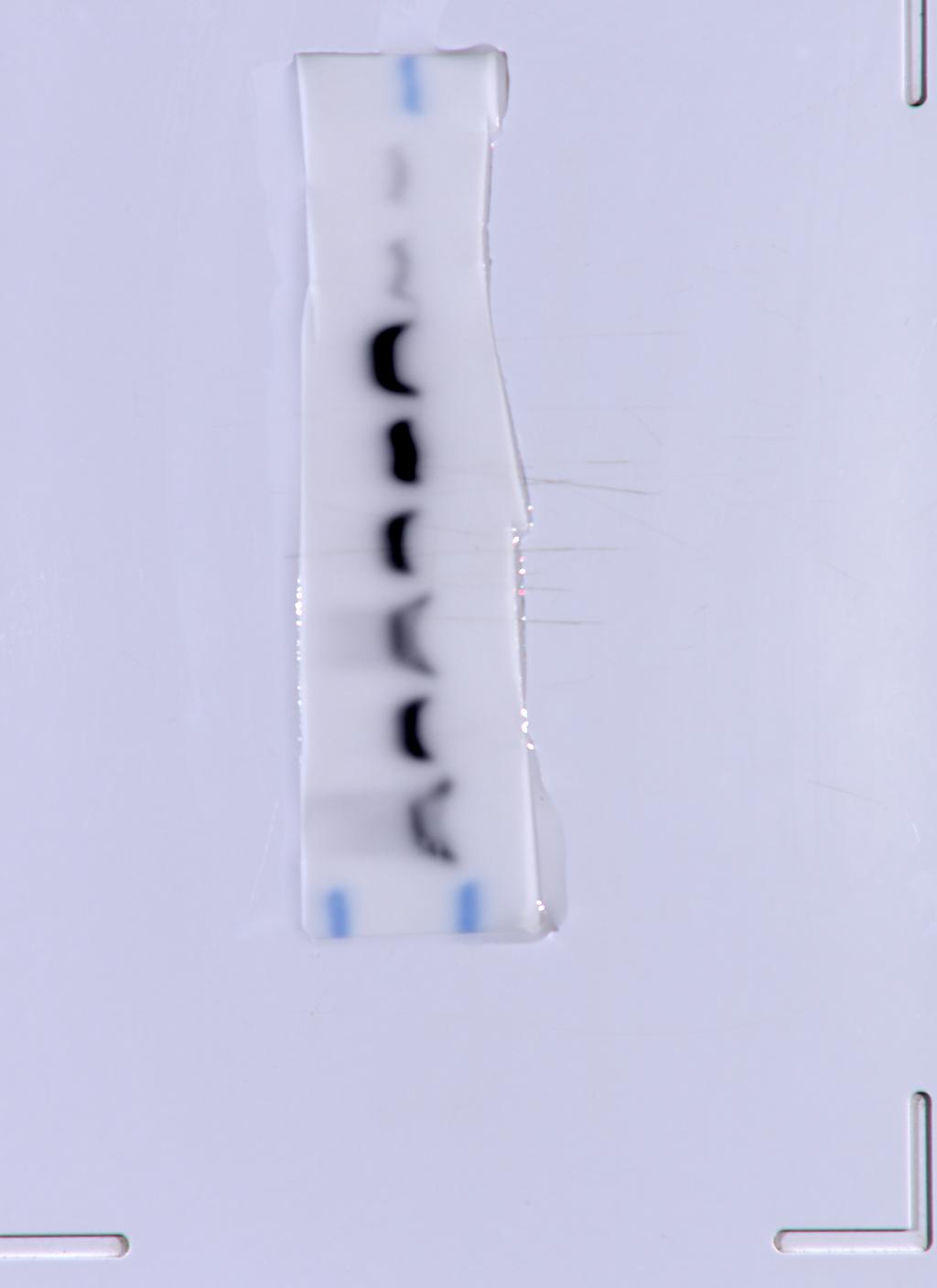

Supplement: Supplementary file 11 — western blot [file 41420_2022_1011_MOESM11_ESM.jpg]

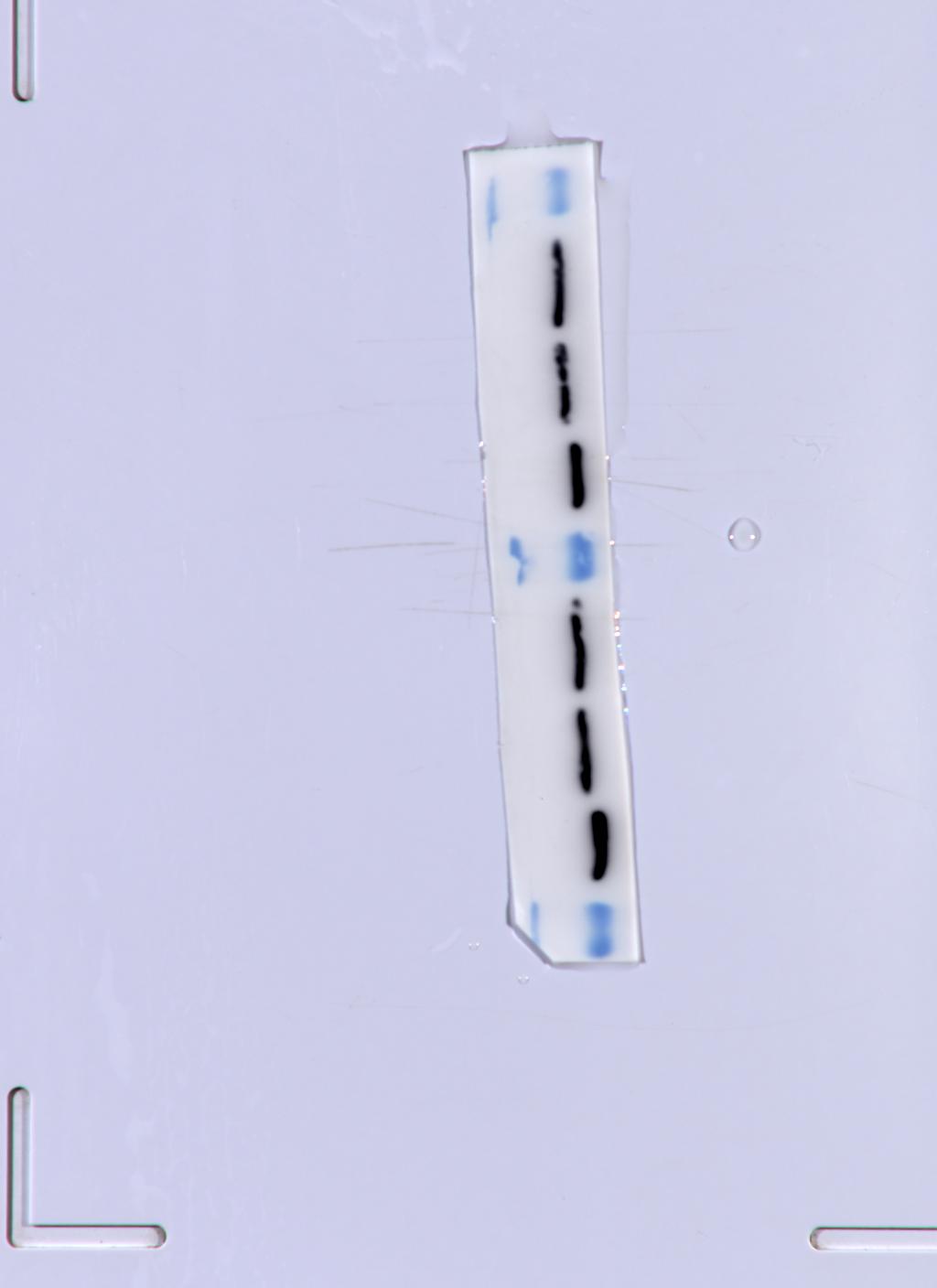

Supplement: Supplementary file 12 — western blot [file 41420_2022_1011_MOESM12_ESM.jpg]

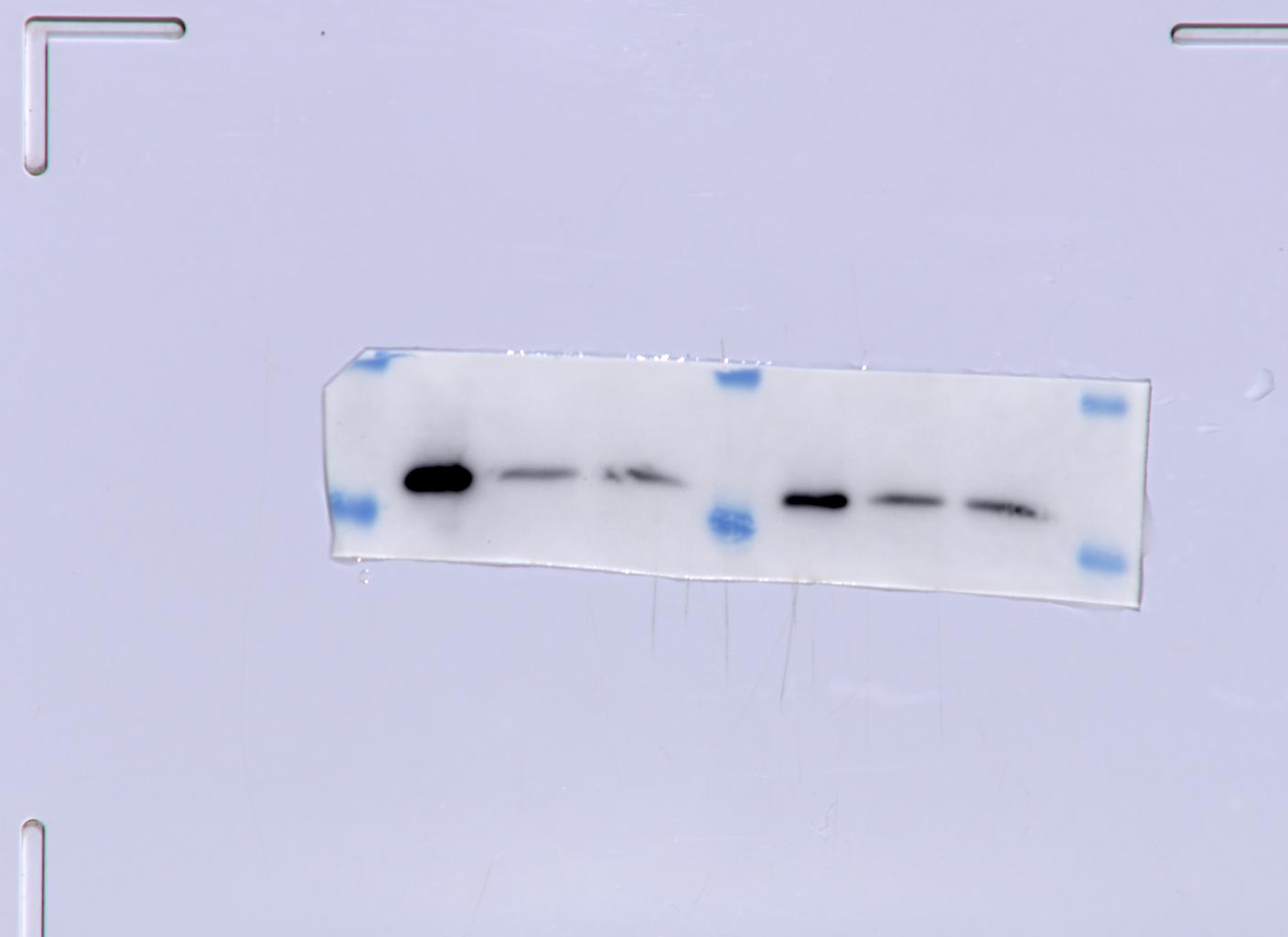

Supplement: Supplementary file 13 — western blot [file 41420_2022_1011_MOESM13_ESM.jpg]

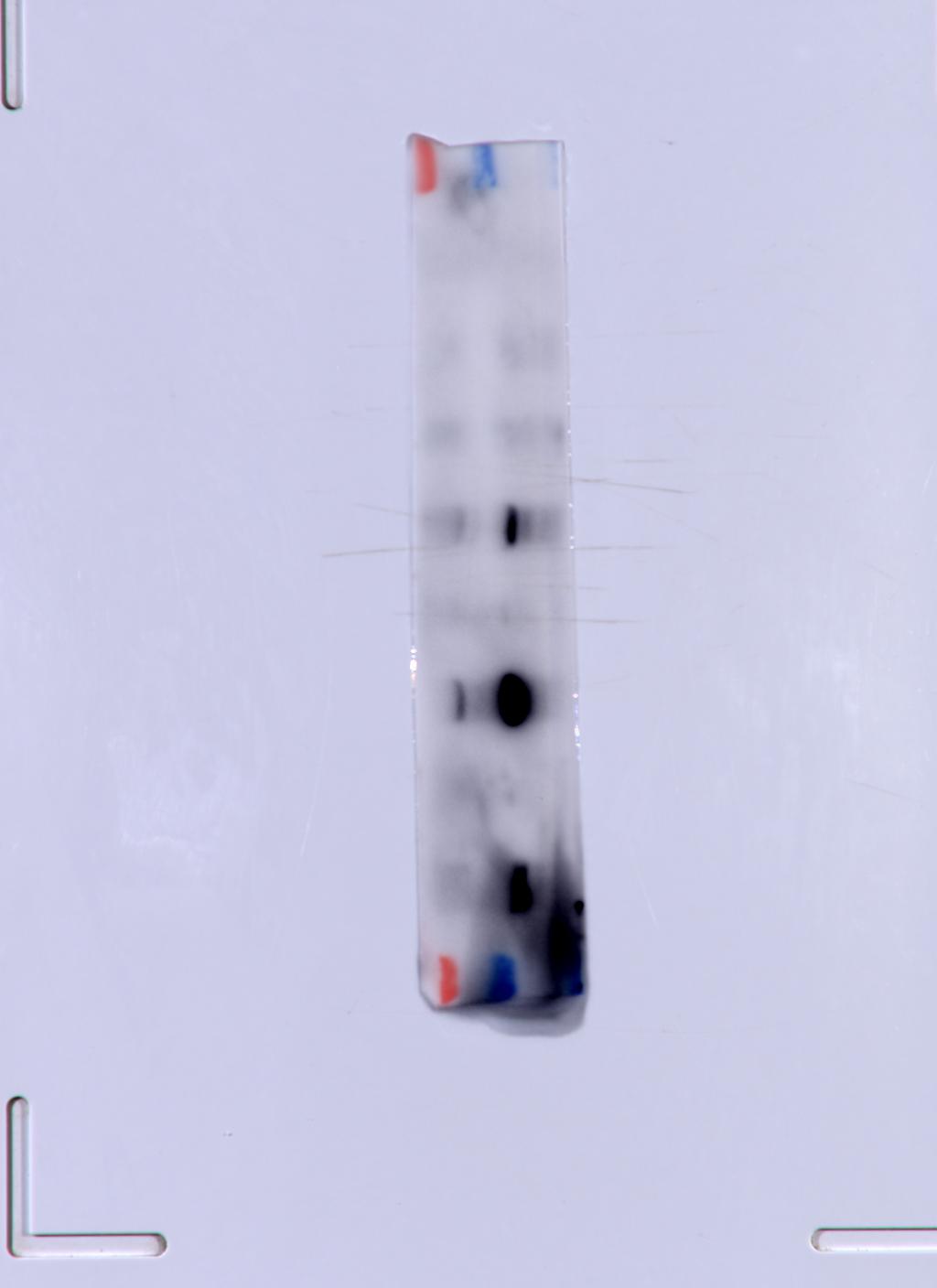

Supplement: Supplementary file 14 — western blot [file 41420_2022_1011_MOESM14_ESM.jpg]

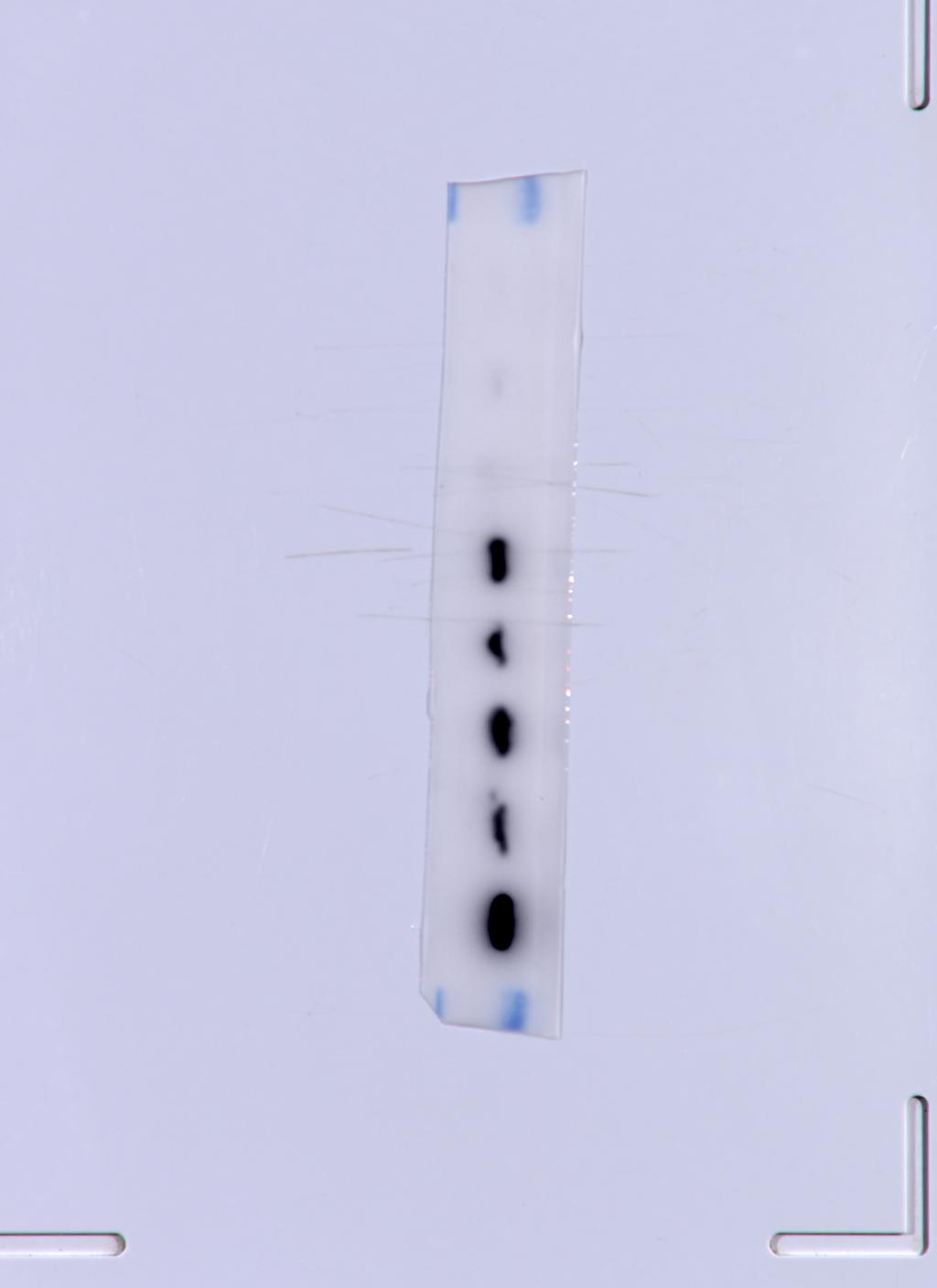

Supplement: Supplementary file 15 — western blot [file 41420_2022_1011_MOESM15_ESM.jpg]

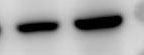

Supplement: Supplementary file 16 — western blot [file 41420_2022_1011_MOESM16_ESM.jpg]

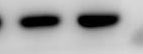

Supplement: Supplementary file 17 — western blot [file 41420_2022_1011_MOESM17_ESM.jpg]

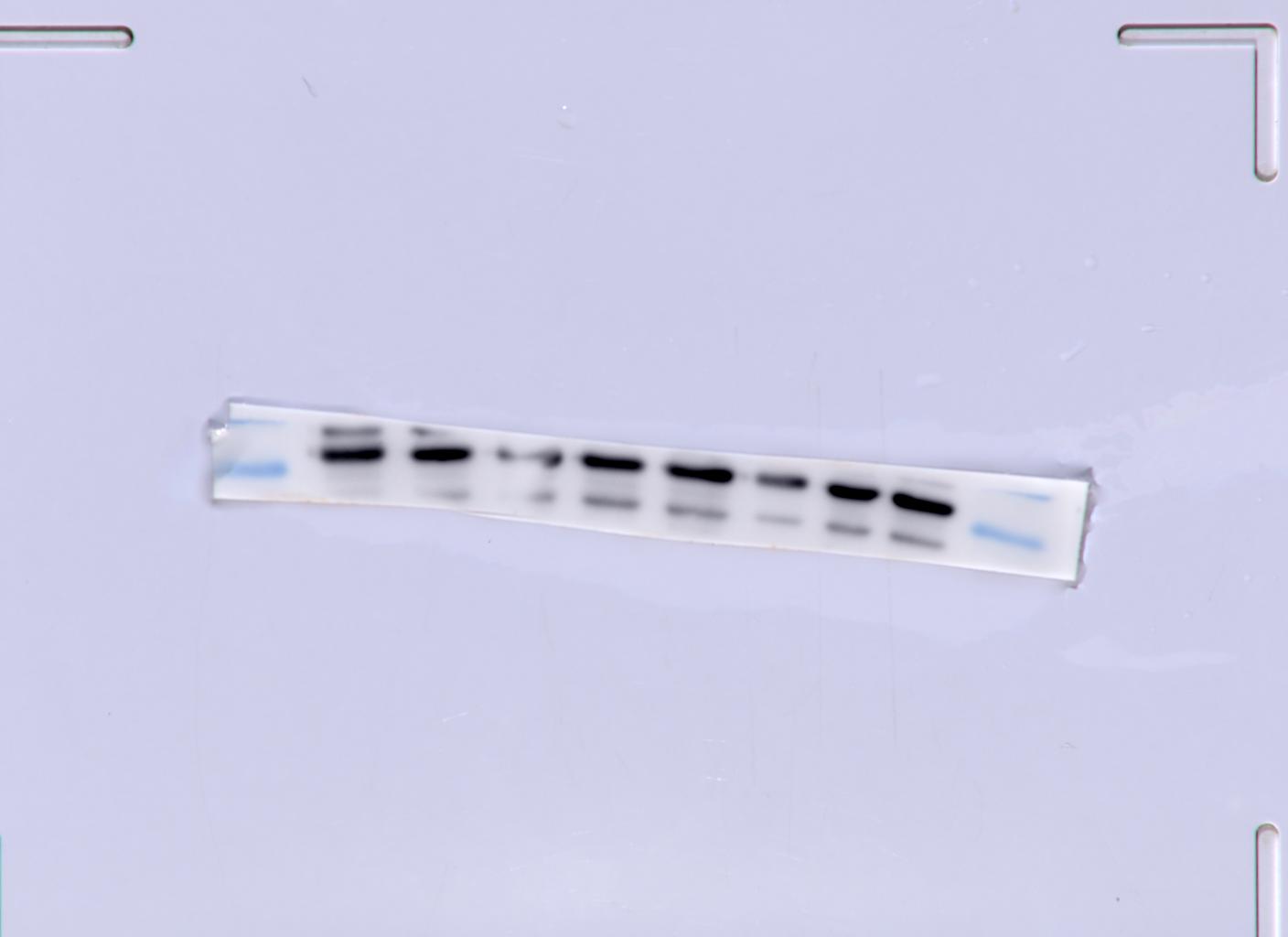

Supplement: Supplementary file 18 — western blot [file 41420_2022_1011_MOESM18_ESM.jpg]

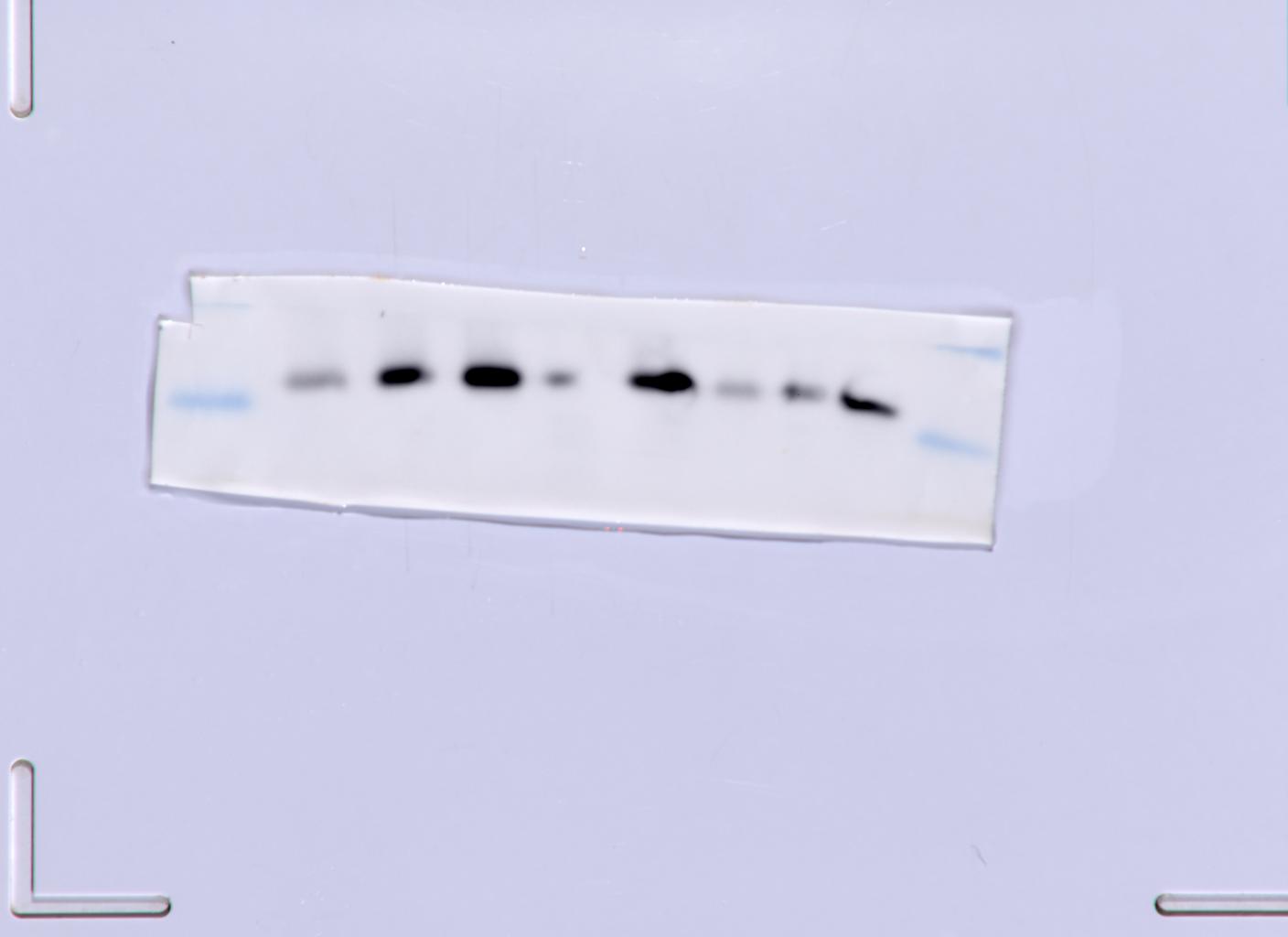

Supplement: Supplementary file 19 — western blot [file 41420_2022_1011_MOESM19_ESM.jpg]

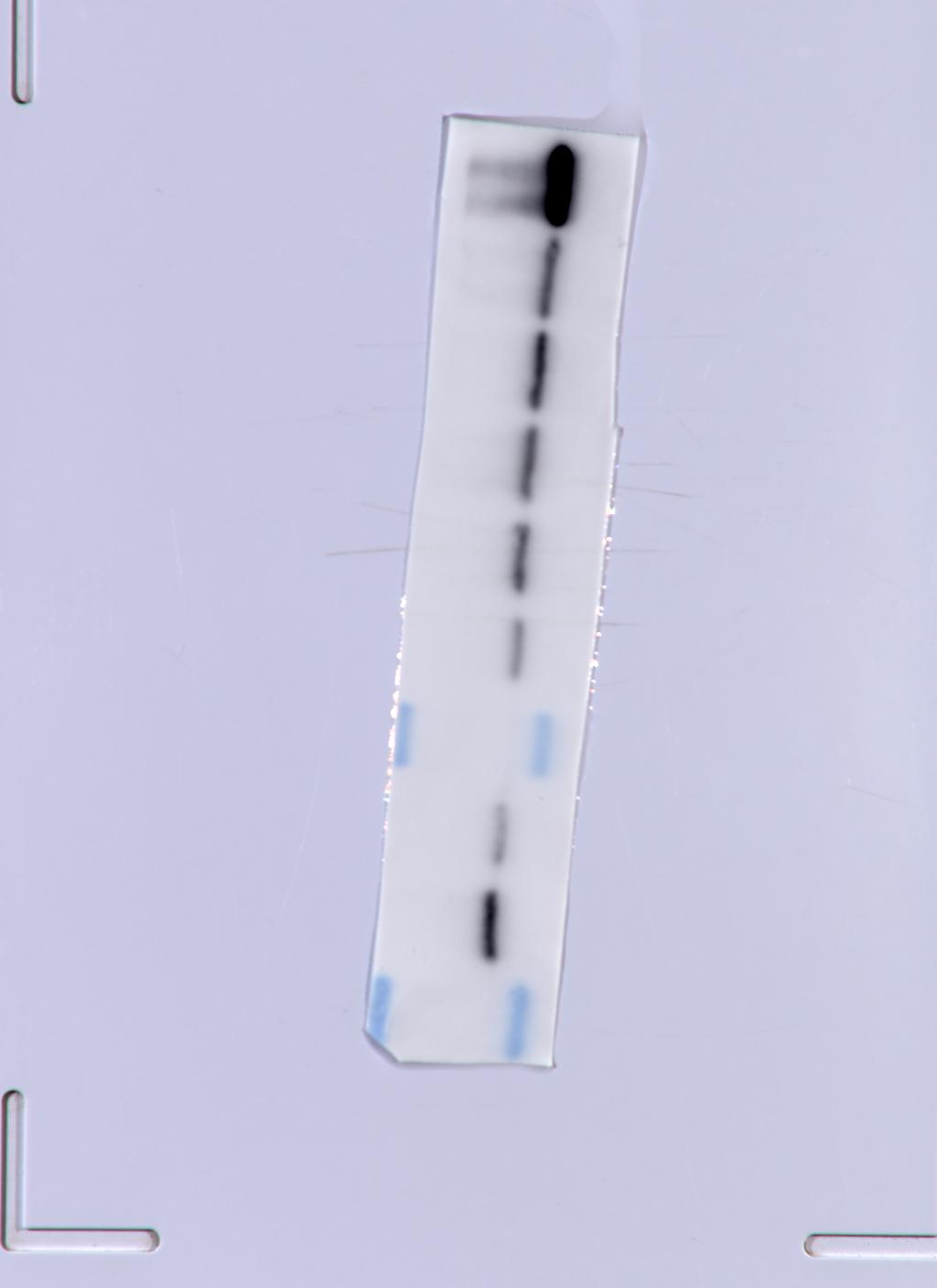

Supplement: Supplementary file 21 — western blot [file 41420_2022_1011_MOESM21_ESM.jpg]

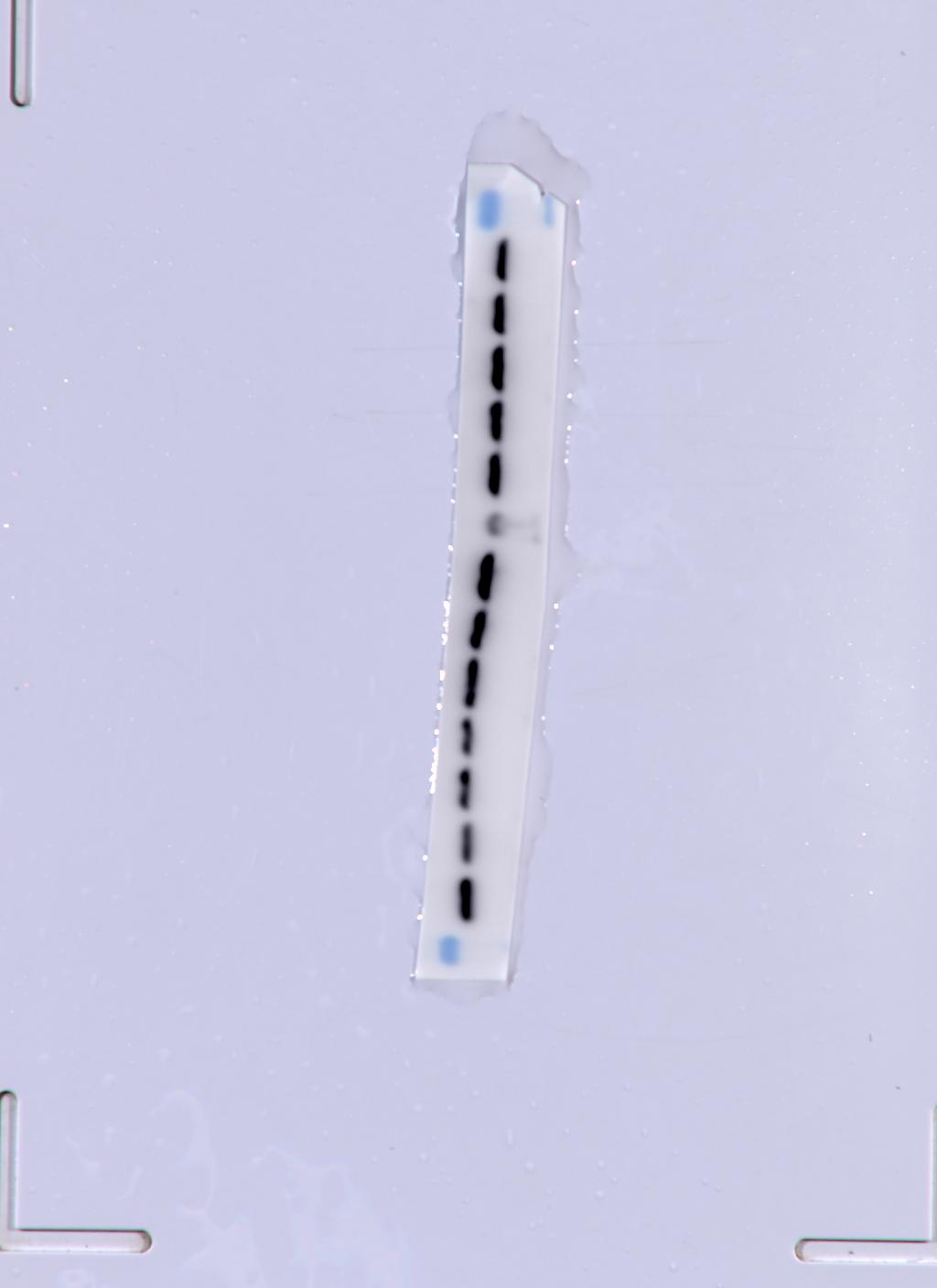

Supplement: Supplementary file 24 — western blot [file 41420_2022_1011_MOESM24_ESM.jpg]

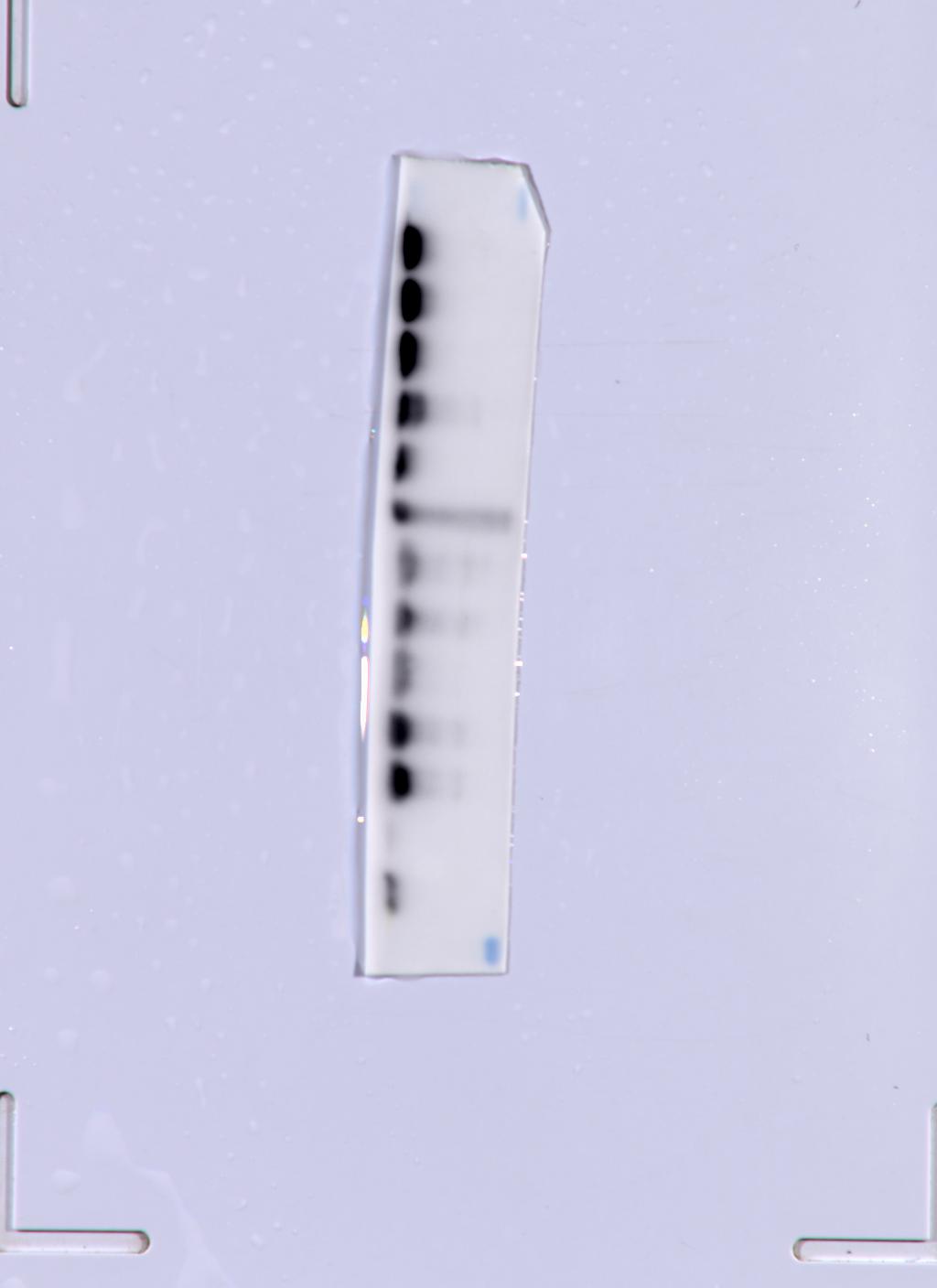

Supplement: Supplementary file 25 — western blot [file 41420_2022_1011_MOESM25_ESM.jpg]

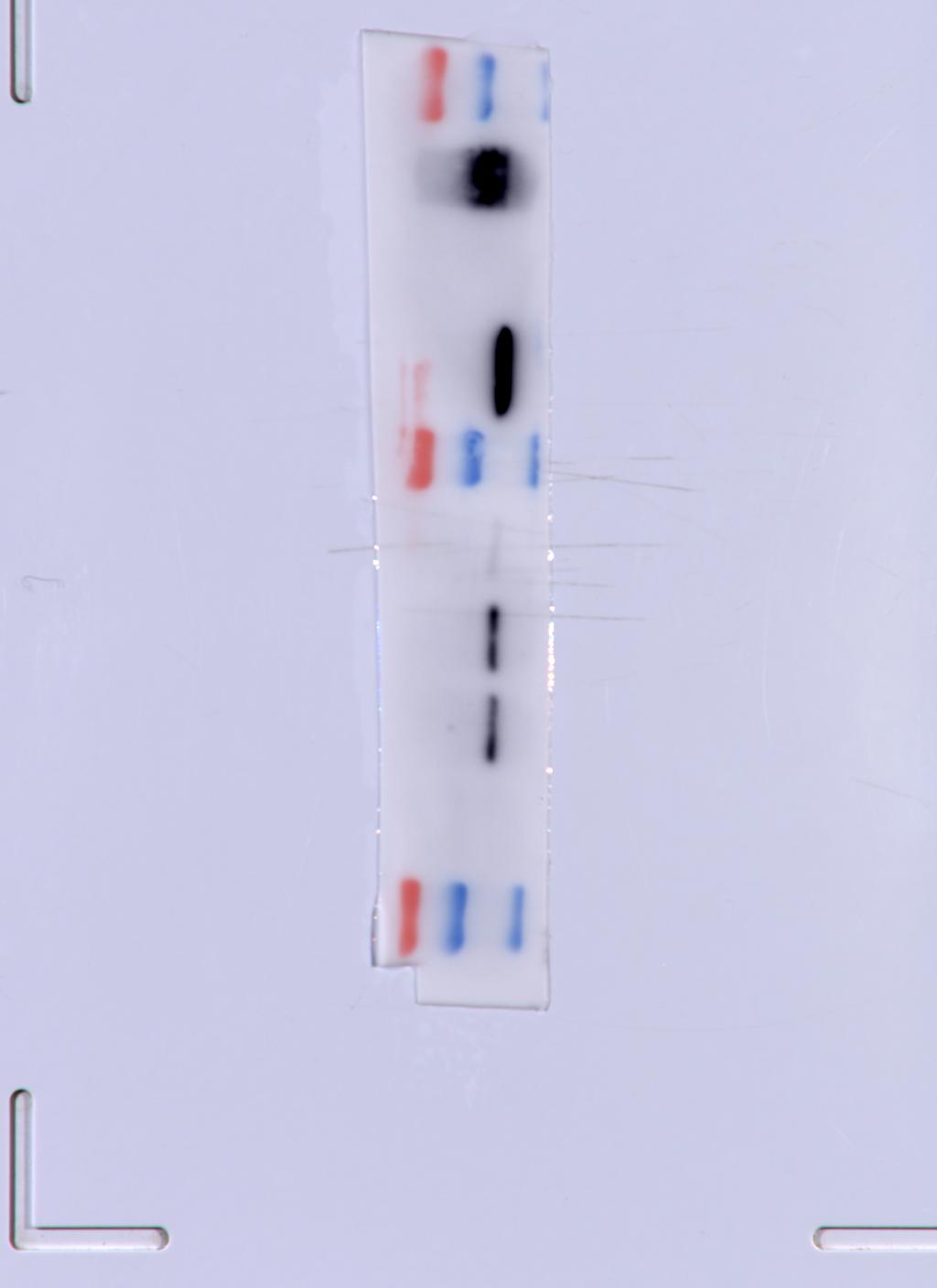

Supplement: Supplementary file 26 — western blot [file 41420_2022_1011_MOESM26_ESM.jpg]

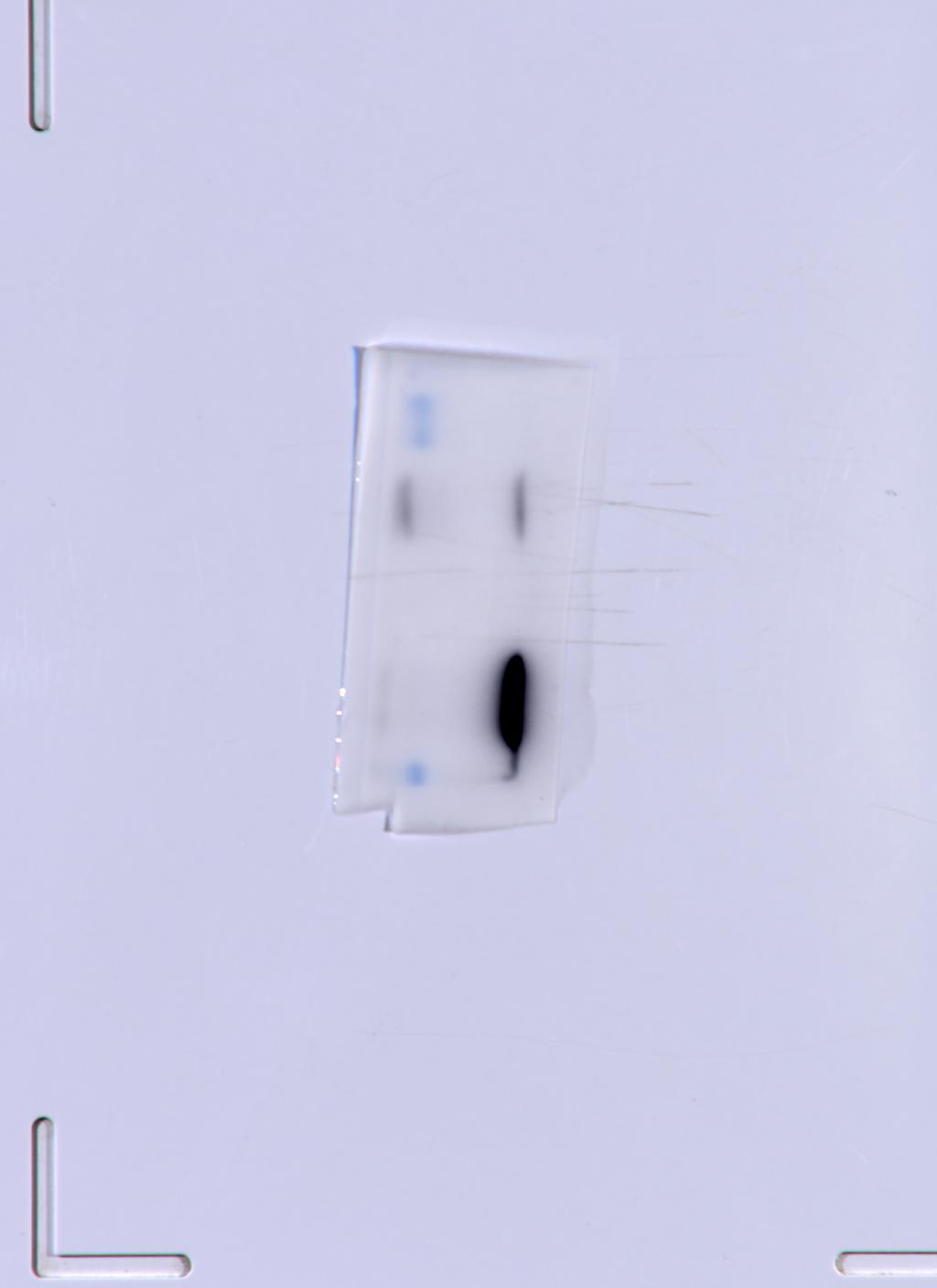

Supplement: Supplementary file 27 — western blot [file 41420_2022_1011_MOESM27_ESM.jpg]

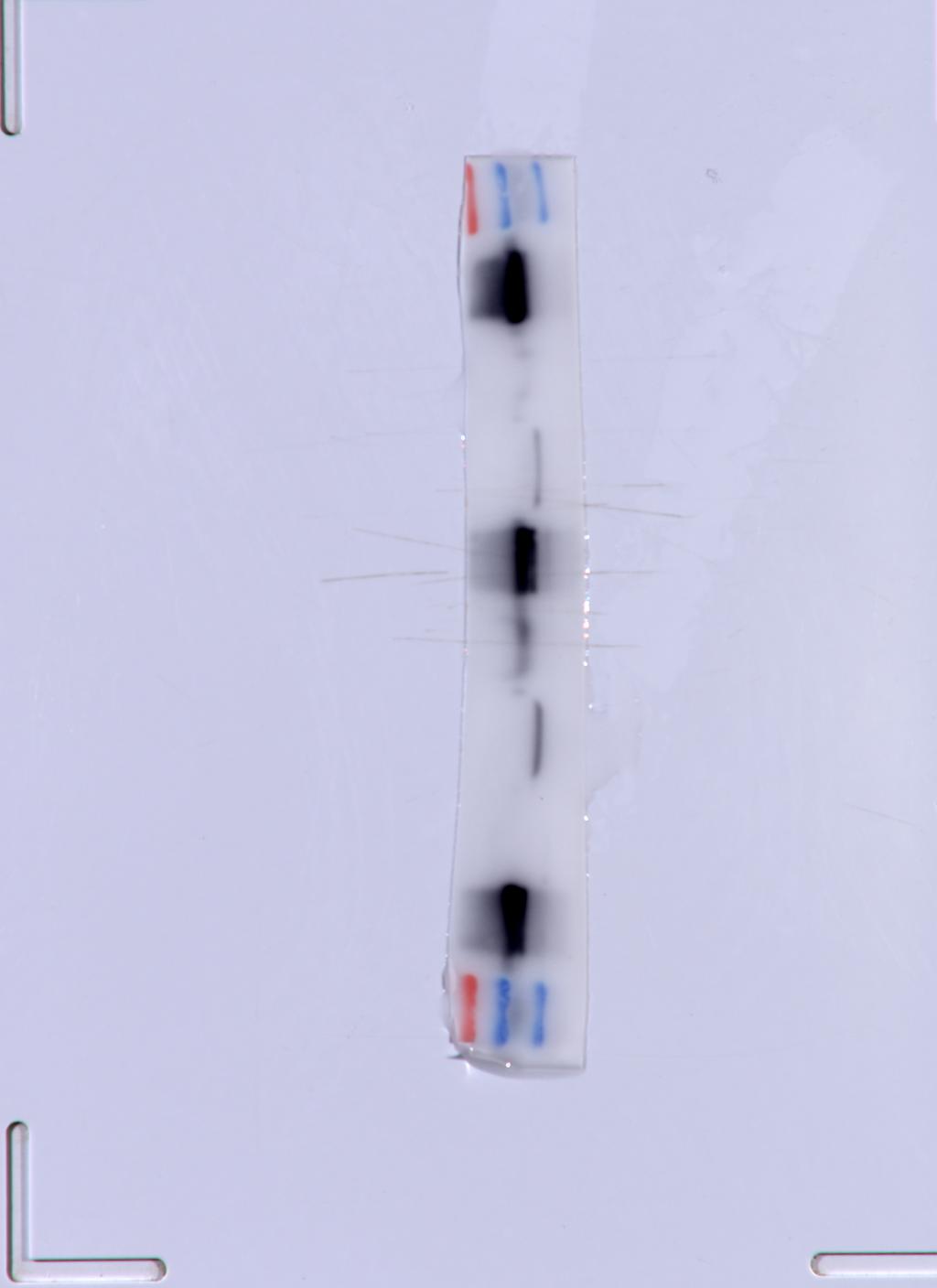

Supplement: Supplementary file 28 — western blot [file 41420_2022_1011_MOESM28_ESM.jpg]

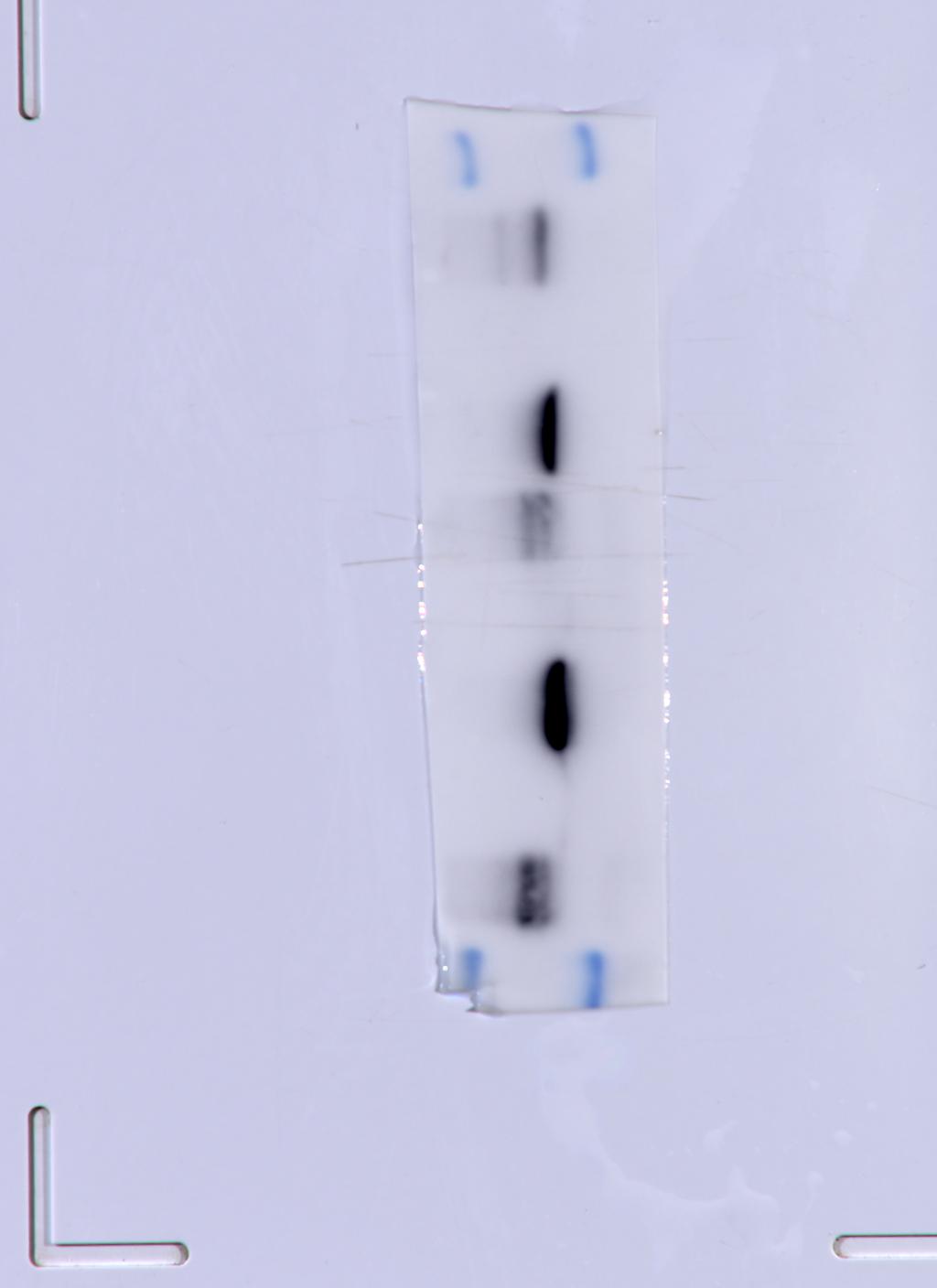

Supplement: Supplementary file 29 — western blot [file 41420_2022_1011_MOESM29_ESM.jpg]

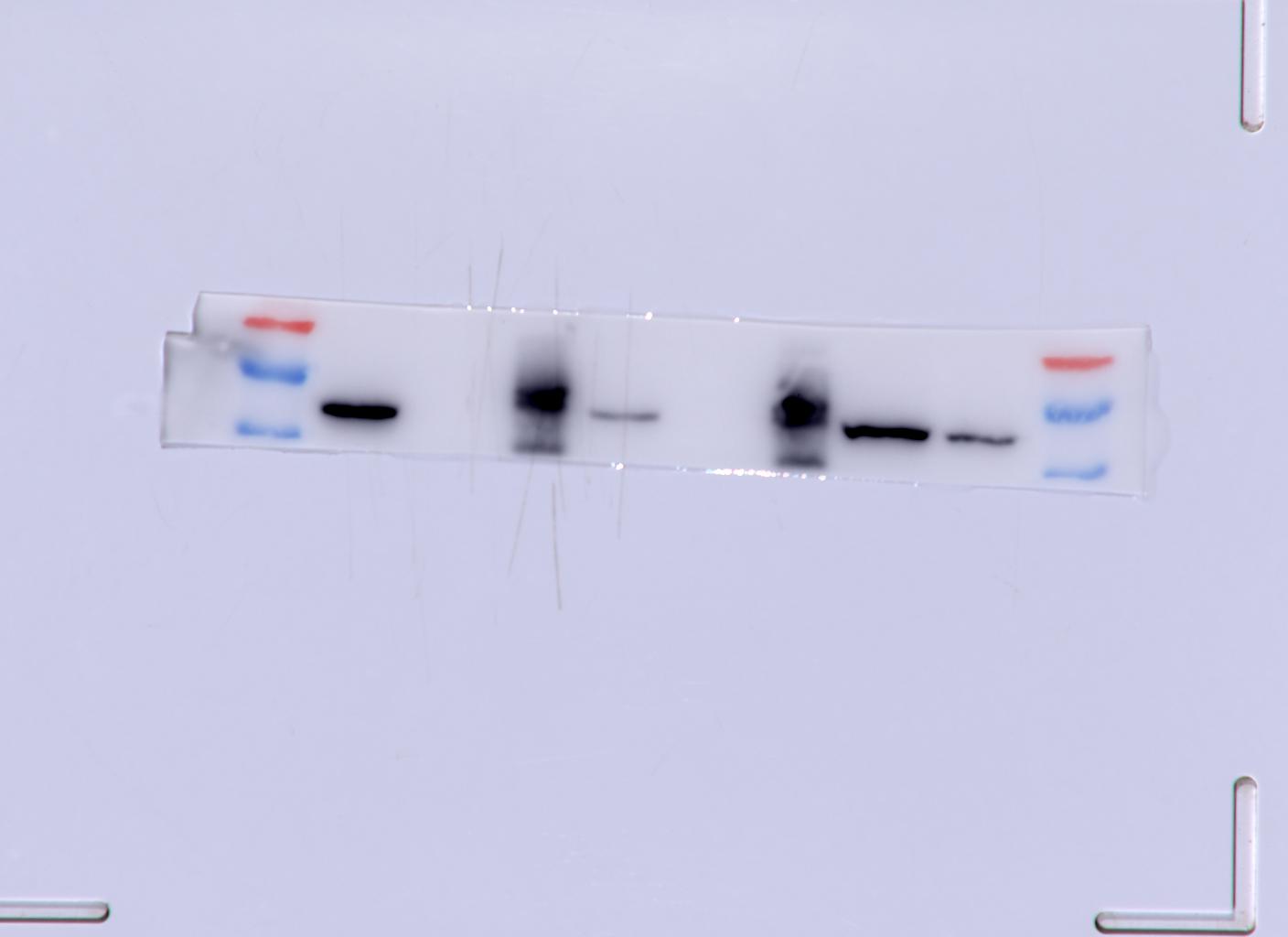

Supplement: Supplementary file 30 — western blot [file 41420_2022_1011_MOESM30_ESM.jpg]

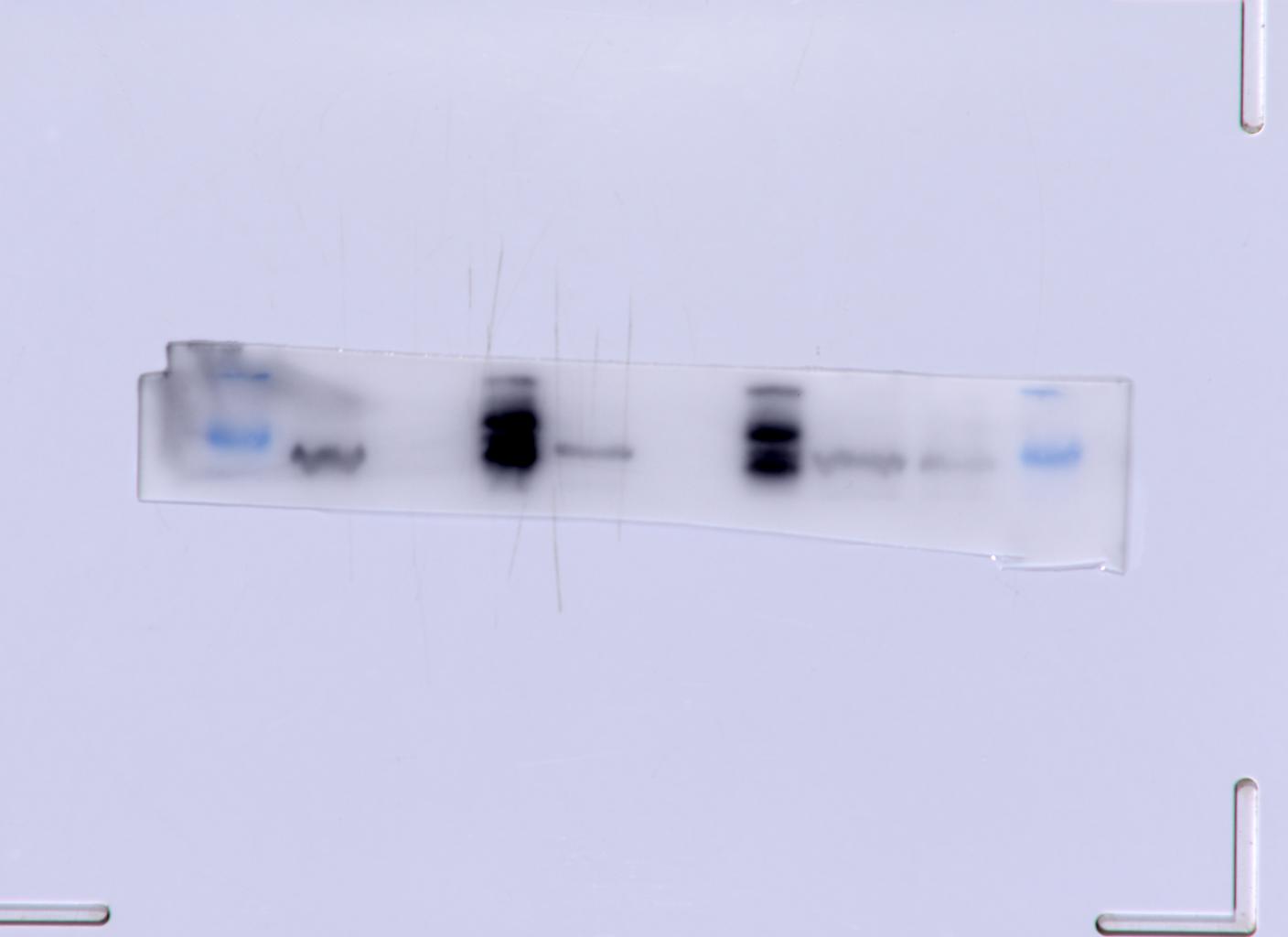

Supplement: Supplementary file 31 — western blot [file 41420_2022_1011_MOESM31_ESM.jpg]

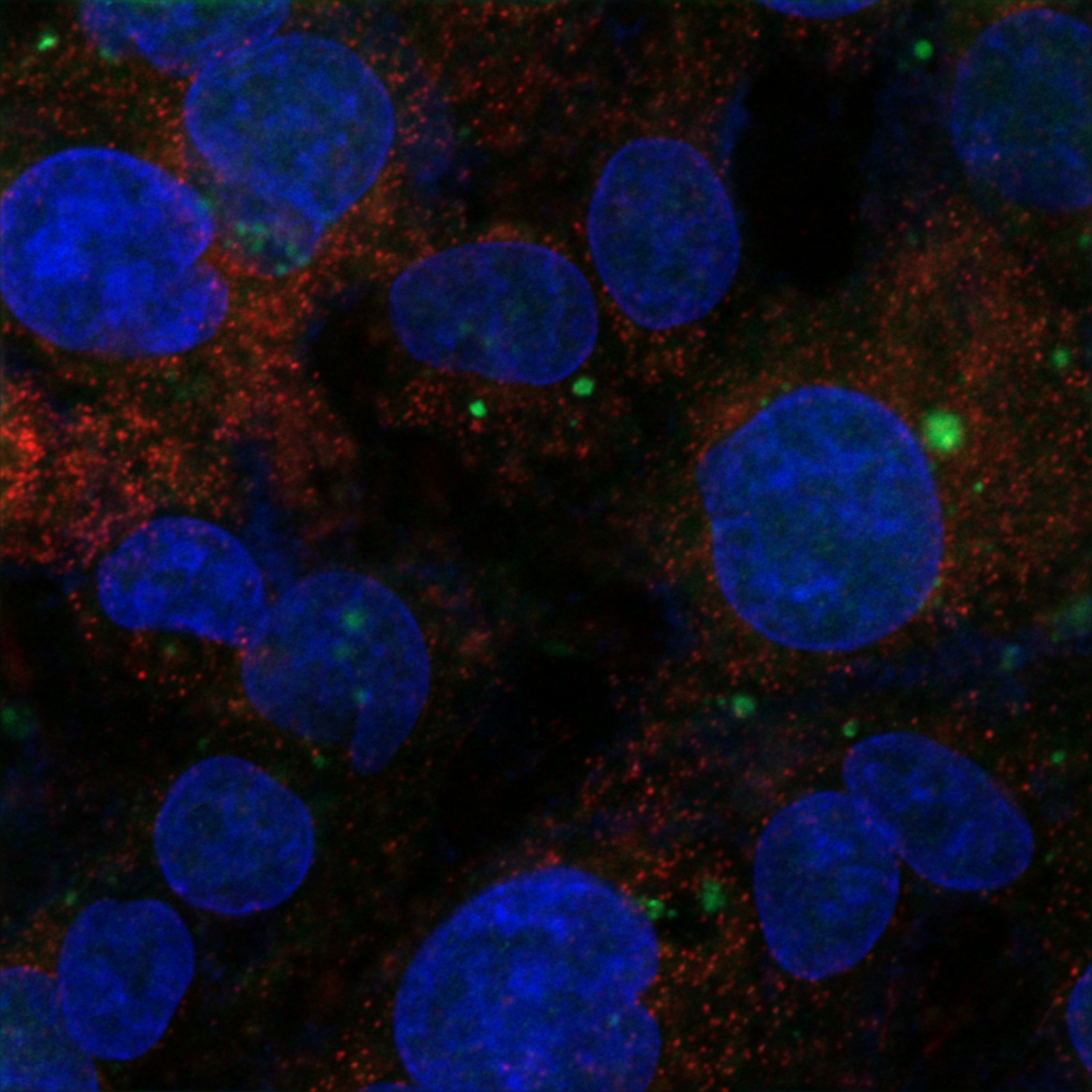

Supplement: Supplementary file 32 — confocal [file 41420_2022_1011_MOESM32_ESM.jpg]

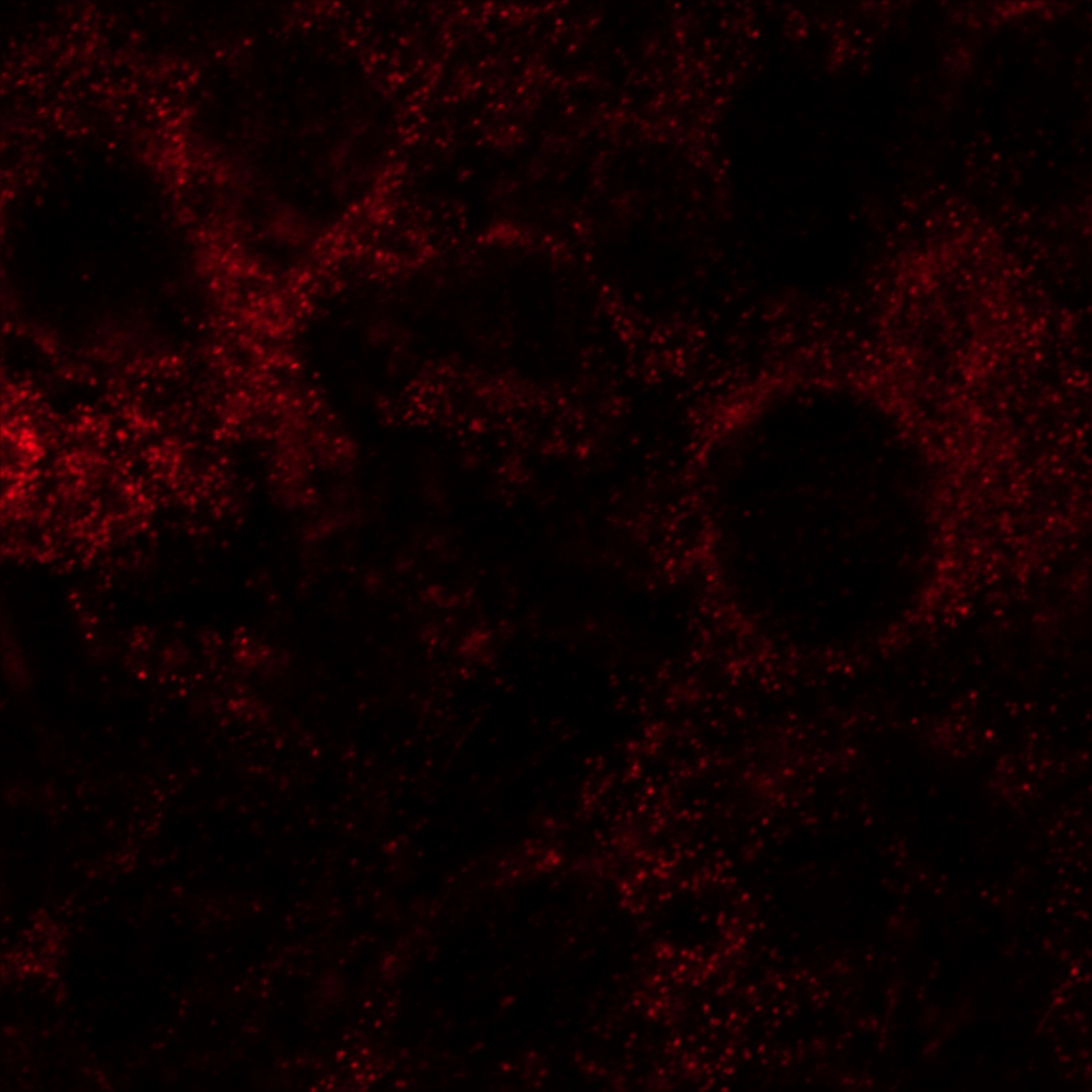

Supplement: Supplementary file 33 — confocal [file 41420_2022_1011_MOESM33_ESM.jpg]

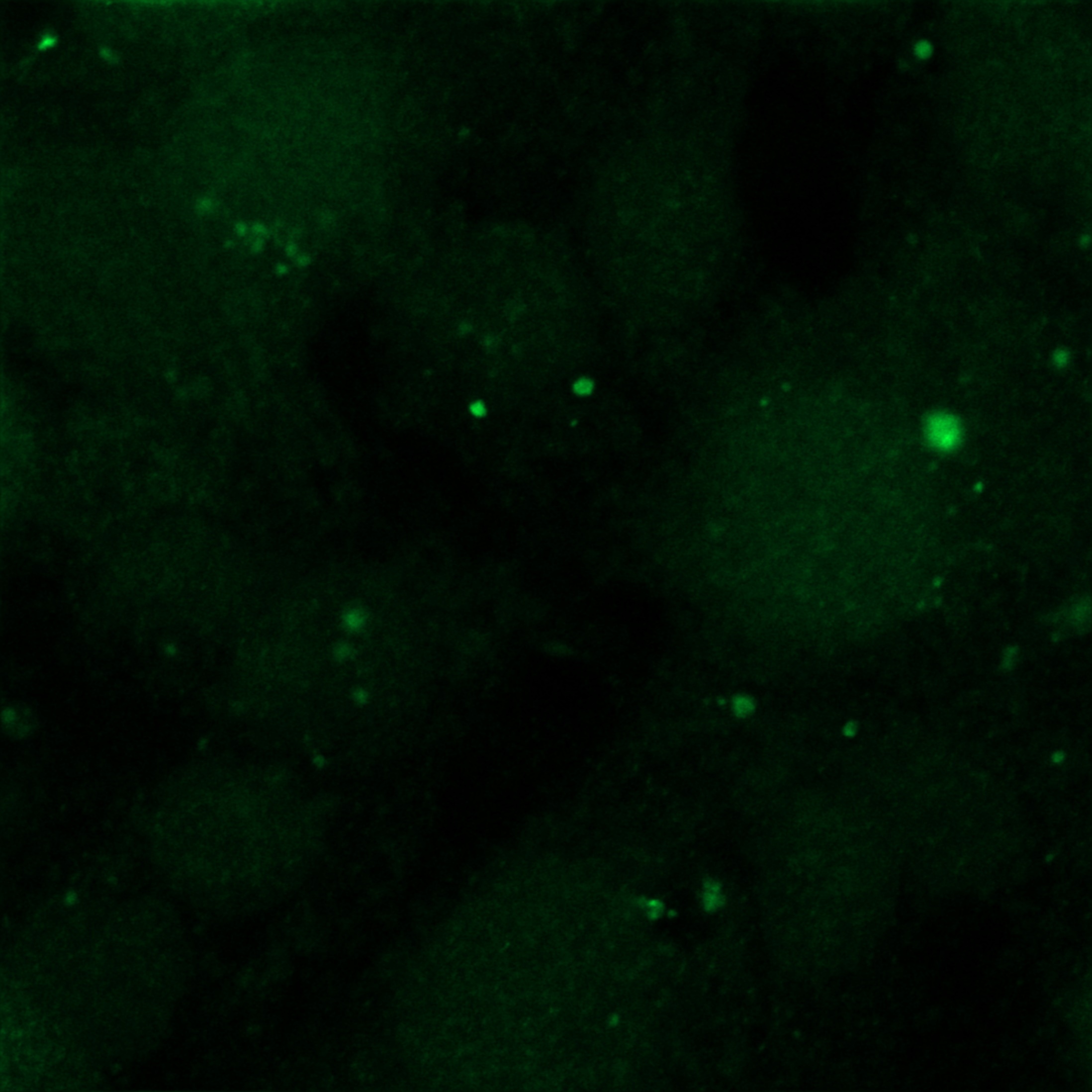

Supplement: Supplementary file 34 — confocal [file 41420_2022_1011_MOESM34_ESM.jpg]

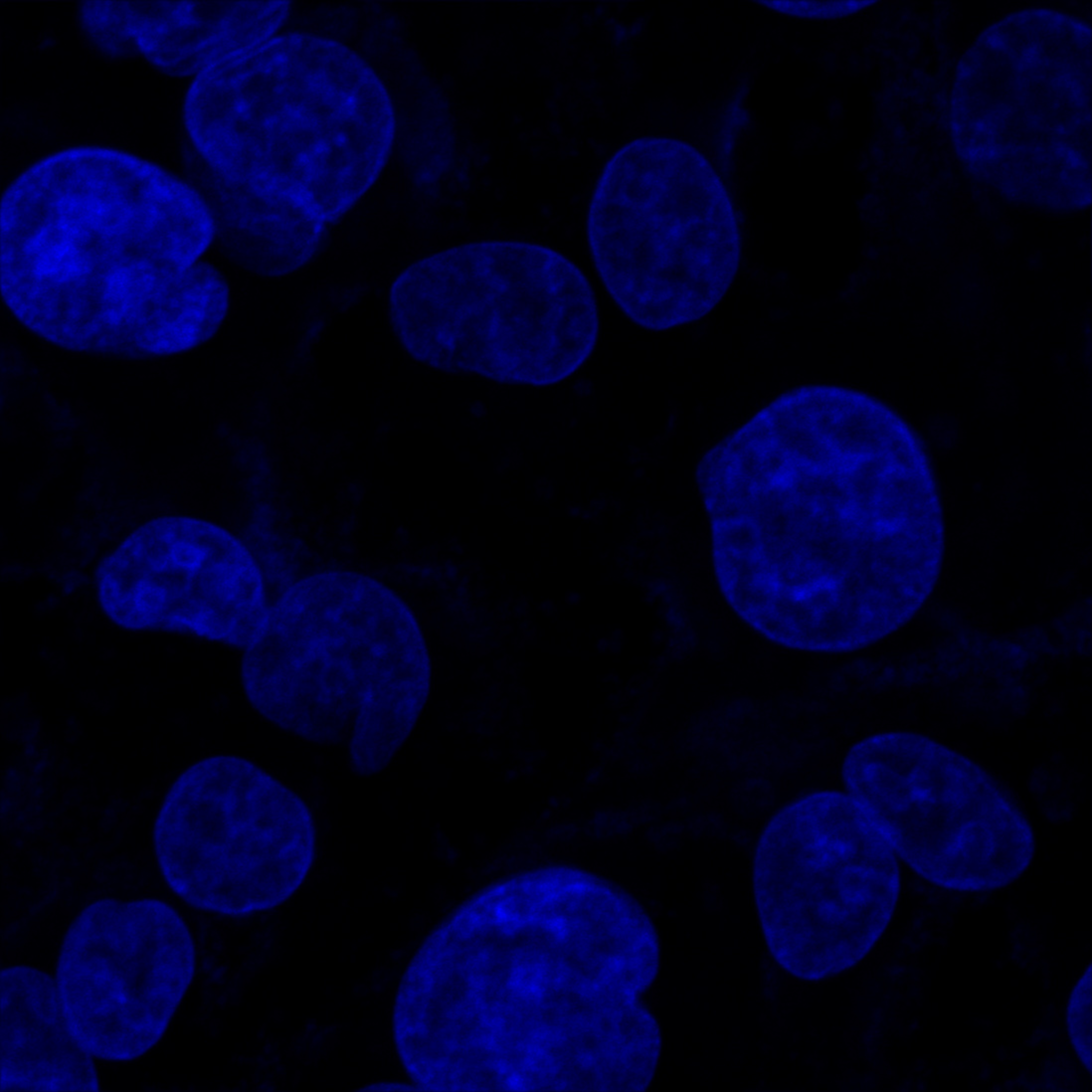

Supplement: Supplementary file 35 — confocal [file 41420_2022_1011_MOESM35_ESM.jpg]

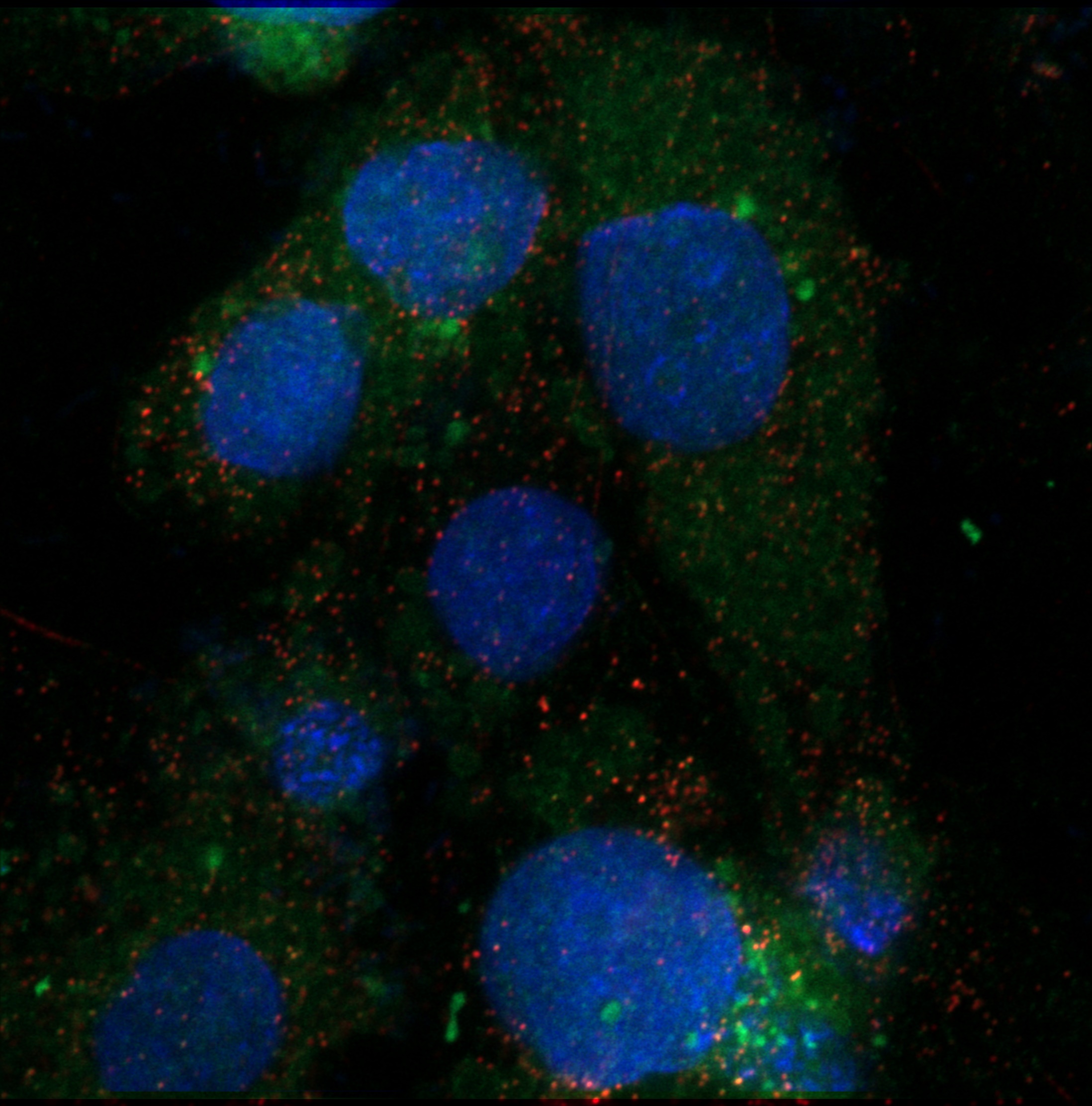

Supplement: Supplementary file 36 — confocal [file 41420_2022_1011_MOESM36_ESM.jpg]

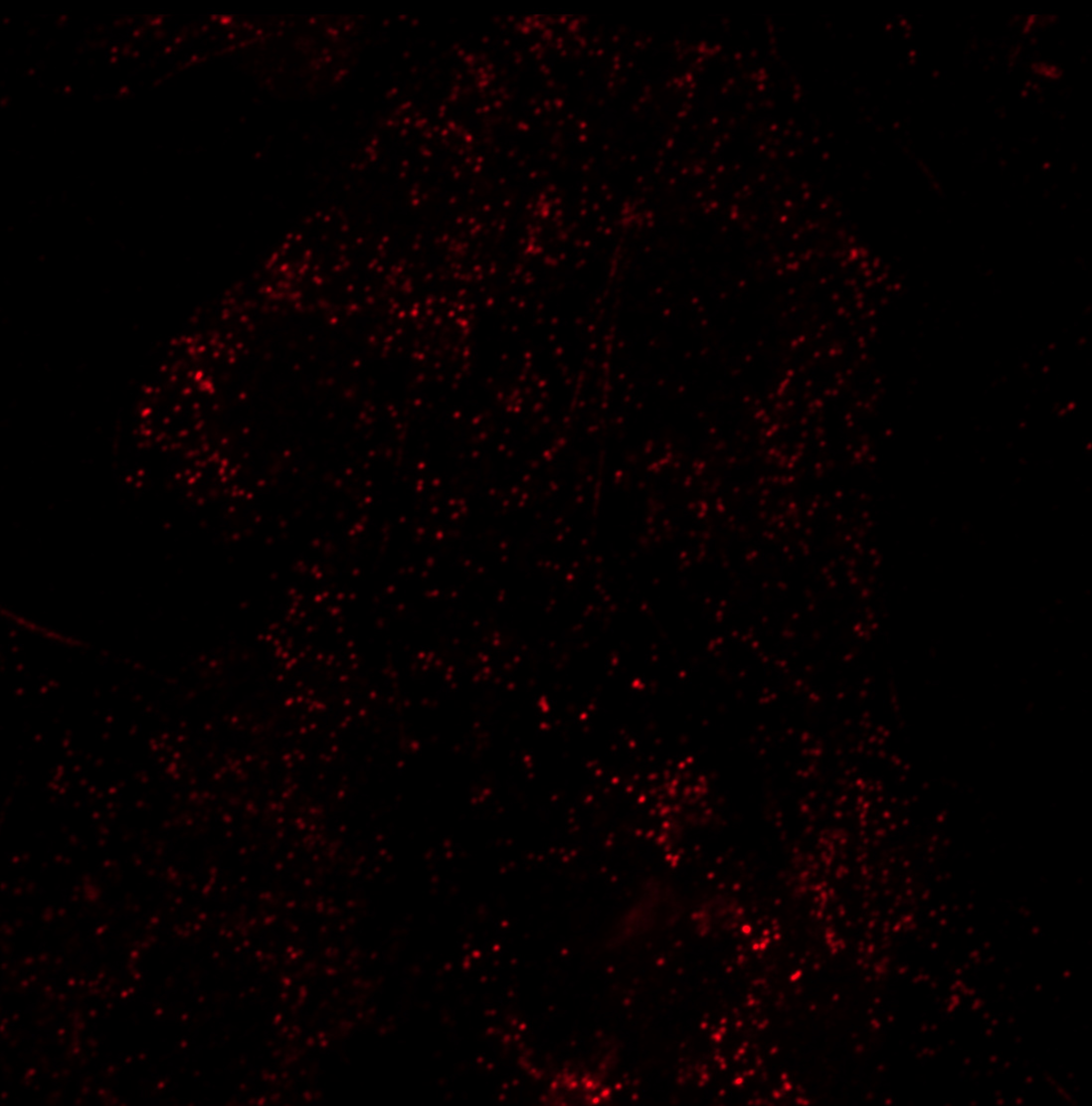

Supplement: Supplementary file 37 — confocal [file 41420_2022_1011_MOESM37_ESM.jpg]

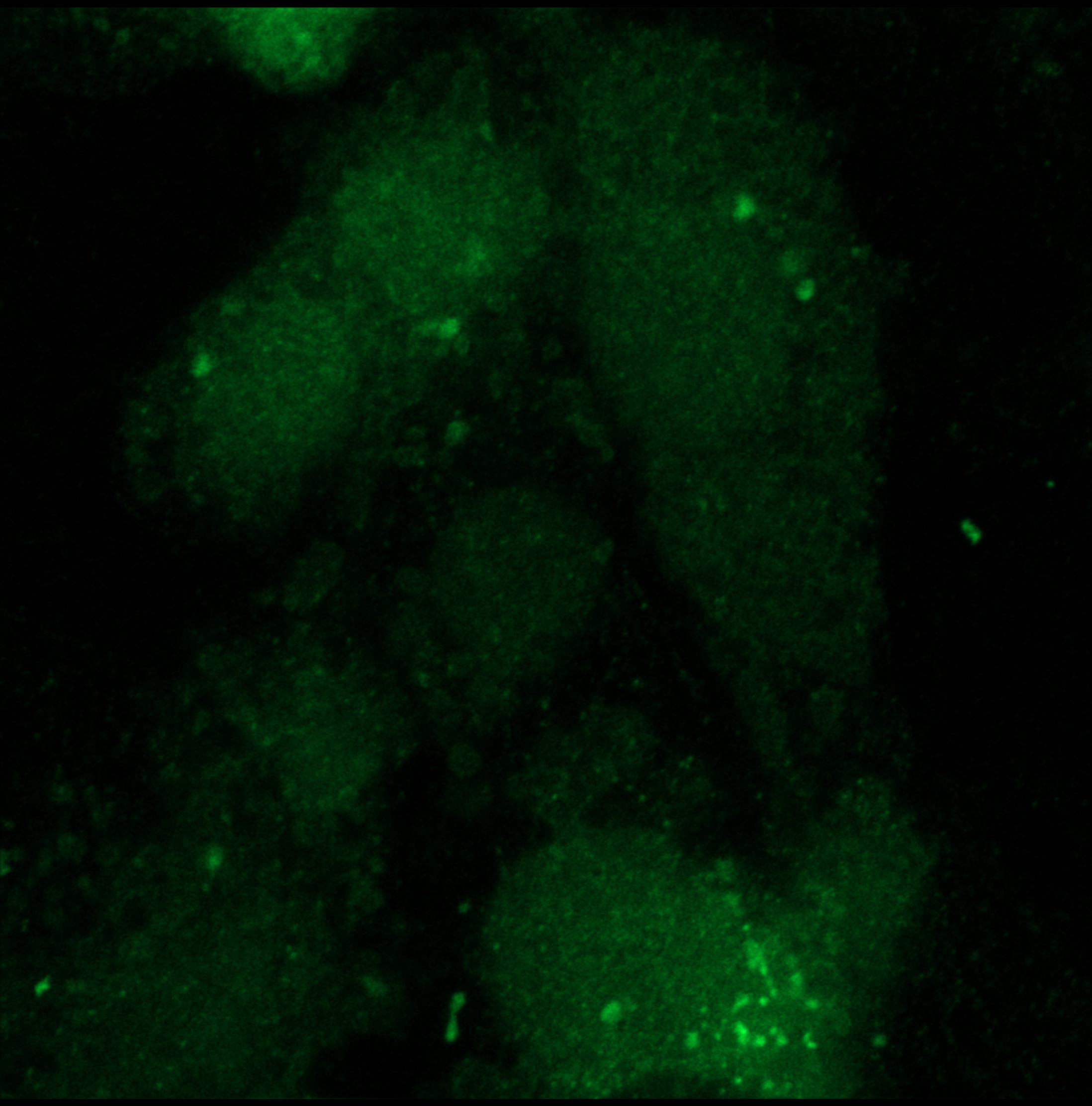

Supplement: Supplementary file 38 — confocal [file 41420_2022_1011_MOESM38_ESM.jpg]

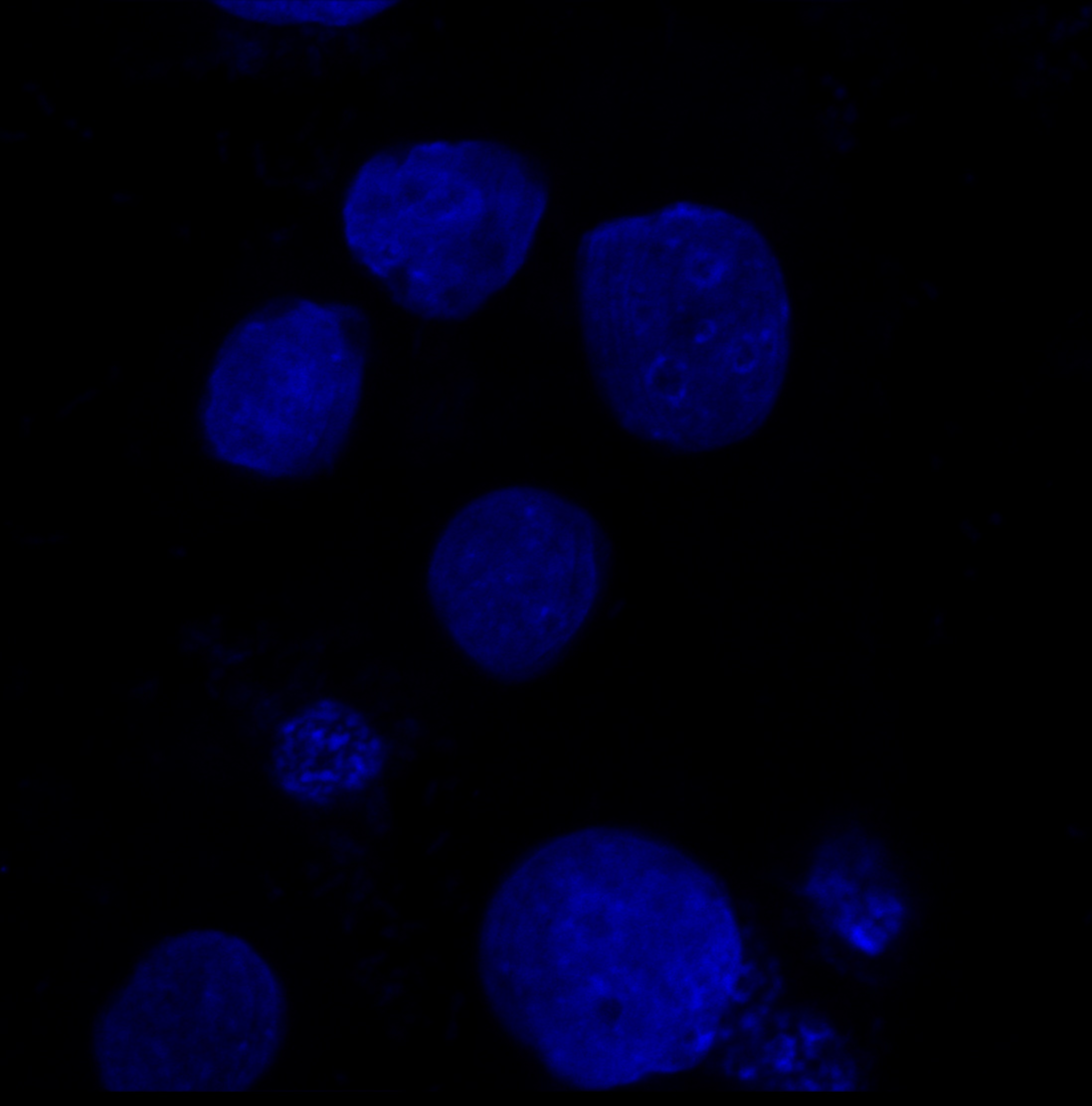

Supplement: Supplementary file 39 — confocal [file 41420_2022_1011_MOESM39_ESM.jpg]

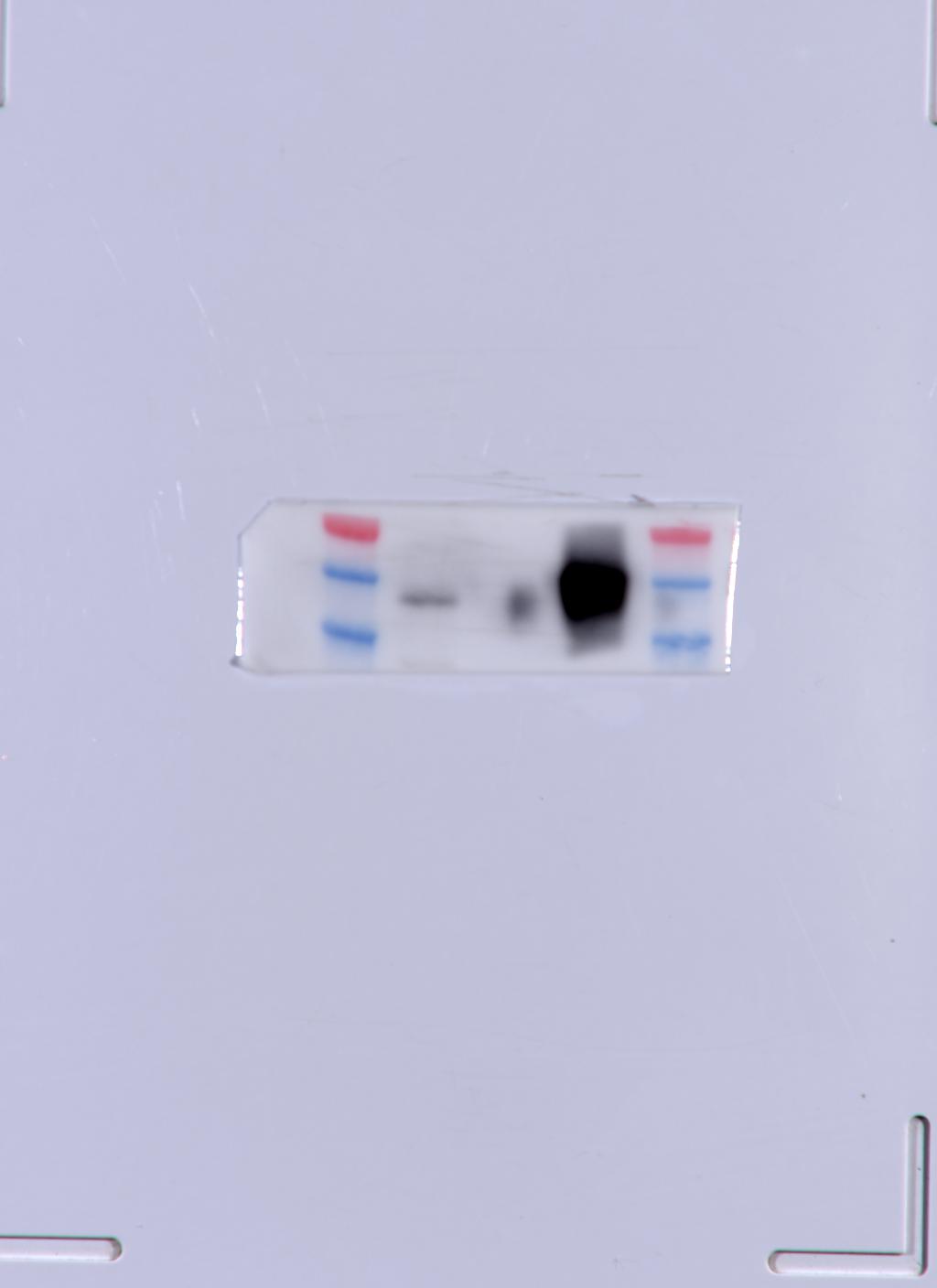

Supplement: Supplementary file 40 — western blot [file 41420_2022_1011_MOESM40_ESM.jpg]
